# Inflammation-driven periostin in ECRS has contrasting effects on tissue structural integrity and osteitis

**DOI:** 10.3389/fimmu.2025.1596746

**Published:** 2025-06-18

**Authors:** Soo-In Kim, Min-Seok Rha, Jinsun Kim, Sang Hyeon Ahn, Ji-Hwan Ryu, Hyung-Ju Cho, Chang-Hoon Kim

**Affiliations:** ^1^ The Airway Mucus Institute, Yonsei University College of Medicine, Seoul, Republic of Korea; ^2^ Department of Biomedical Sciences, Yonsei University College of Medicine, Seoul, Republic of Korea; ^3^ Brain Korea 21 PLUS Project for Medical Science, Yonsei University College of Medicine, Seoul, Republic of Korea; ^4^ Department of Otorhinolaryngology, Yonsei University College of Medicine, Seoul, Republic of Korea; ^5^ Human Microbiome Center, Yonsei University College of Medicine, Seoul, Republic of Korea; ^6^ Department of Otorhinolaryngology, Bundang Jesaeng Hospital, Seongnam, Republic of Korea

**Keywords:** periostin, Th2 inflammation, eosinophilic chronic rhinosinusitis, osteitis, tissue remodeling

## Abstract

**Introduction:**

Eosinophilic chronic rhinosinusitis (ECRS) is a severe form of chronic rhinosinusitis characterized by type 2 inflammation, tissue remodeling, and bone thickening, known as osteitis. Periostin, a matricellular protein involved in extracellular matrix (ECM) regulation and T helper 2 (Th2)-mediated inflammation, is markedly elevated in patients with ECRS; however, its pathophysiological role remains unclear.

**Methods:**

We investigated the role of periostin in inflammation and tissue remodeling in ECRS using samples from ECRS patients, human nasal epithelial cells and fibroblasts, as well as an ECRS mouse model including periostin knockout mice.

**Results:**

Periostin levels were elevated in ECRS tissues and modestly correlated with osteitis scores. Th2 cytokines increased periostin expression, particularly in nasal fibroblasts. Conditioned medium containing periostin promoted osteogenic differentiation *in vitro*, whereas neutralizing antibodies reduced the expression of osteogenic markers. In an ECRS mouse model, periostin deficiency led to reduced bone thickening and lower expression of osteogenic markers despite similar eosinophil infiltration. Furthermore, periostin-deficient mice exhibited greater epithelial collapse and reduced fibronectin levels, indicating compromised ECM integrity.

**Discussion:**

These findings demonstrate that periostin contributes to osteogenesis and maintenance of structural stability in the inflamed sinonasal mucosa. Periostin may be a potential therapeutic target for controlling chronic inflammation and tissue remodeling in ECRS.

## 1 Introduction

Periostin (also known as osteoblast-specific factor 2), a member of the matricellular protein family, has been identified as a key contributor to various biological processes, including tissue remodeling, metastasis, and cancer (1–3). It also plays a central role in collagen formation, fibrosis, epithelial-mesenchymal transition, and T helper (Th)2-driven inflammation, making it a biomarker for diseases such as cancer, asthma, and idiopathic pulmonary fibrosis (4–6). Various cytokines, including interleukin (IL)-4, IL-13, IL-5, and bone morphogenetic protein (BMP)-2, regulate periostin expression (7–11).

Beyond its role as a disease marker, periostin actively participates in tissue remodeling through complex interactions with extracellular matrix (ECM) components and multiple cell types (12). It interacts with ECM proteins, such as tenascin and fibronectin, and integrins, specifically α_v_β_3_ and α_v_β_5_. These integrin interactions activate downstream signaling pathways, including PI3K/Akt, FAK, and ERK/MAPK, which promote inflammation, ECM production, and phenotypic changes, such as myofibroblast differentiation, enhanced cell migration, and epithelial-mesenchymal transition (13). Periostin also interacts with the transforming growth factor-beta (TGF-β) signaling pathway to facilitate inflammation and ECM remodeling, thereby advancing fibrosis development (8). In addition, periostin recruits eosinophils and promotes their infiltration, contributing to eosinophil-associated inflammatory diseases, such as bronchial asthma and eosinophilic chronic rhinosinusitis (ECRS) (14, 15).

Periostin is involved in multiple aspects of ECRS and plays a significant role in both inflammation and remodeling. It participates in various phases of eosinophilic inflammation and contributes to disease severity (16). Previous studies have reported that, in patients with ECRS, periostin expression is associated with increased levels of Th2 inflammatory markers, including IL-5, eotaxin-3, and fractional exhaled nitric oxide (FeNO). Moreover, elevated periostin levels in the blood and tissues correlate with eosinophil counts and infiltration, highlighting its role in regulating eosinophilic activity (17–19). In the lower airway mucosa, periostin promotes ECM remodeling through interactions with fibronectin and tenascin-C, facilitating collagen synthesis, subepithelial fibrosis, and tissue restructuring (9, 20).

In addition to its role in inflammation and tissue remodeling, periostin has been implicated in bone changes associated with chronic rhinosinusitis (CRS), specifically in osteitis and osteoneogenesis. Osteitis refers to inflammation-induced thickening of pre-existing bone, whereas osteoneogenesis refers to the pathological formation of new bone. Chronic Th2-driven inflammation trigger osteitis, which in turn induces osteoneogenesis as part of a remodeling response. These bone changes are associated with CRS severity, as evaluated by endoscopic and radiologic assessments, including computed tomography (CT) and olfactory function scores (21). The osteitis score, determined through CT imaging, correlates with tissue and serum eosinophilia in patients with CRS (22, 23). Elevated periostin expression in CRS has been associated with eosinophilic inflammation and ECM remodeling, indicating that periostin may contribute to structural changes, including osteitic alterations (17). Therefore, it may promote abnormal bone restructuring, resulting from sustained inflammation. However, its pathophysiological role remains unclear.

In this study, we investigated the cellular origin of periostin in the sinonasal mucosa and explored the relationship between Th2 cytokine-induced periostin secretion and osteitis in patients with ECRS. Furthermore, we examined the effects of Th2 inflammation-driven periostin on chronic inflammation and tissue remodeling in patients with ECRS.

## 2 Materials and methods

### 2.1 Human ethmoid mucosa

Human nasal ethmoid mucosa samples were obtained from 26 patients. Samples from 23 patients diagnosed with CRS (10 with ECRS and 13 with non-ECRS) were collected during endoscopic sinus surgery. Control samples (n = 3) were obtained from anatomically normal ethmoid mucosa of patients undergoing surgery for septal deviation or maxillary cancer. Detailed demographic and clinical information of the patients are provided in **Supplementary Table S1**. The tissue samples were equally divided. Half of the samples were fixed overnight in 4% paraformaldehyde and embedded in paraffin. The other half were prepared for protein extraction by freezing with liquid nitrogen, grinding, and mixing with RIPA buffer (Sigma) and a protease inhibitor cocktail, followed by incubation on ice for 1 h. The samples were centrifuged at 12,000 rpm for 15 min at 4°C, and the supernatant (lysate) was transferred into a fresh tube. This study was reviewed and approved by the Institutional Review Board of Severance Hospital (No. 4-2021-0573) and conducted in accordance with the principles of the Declaration of Helsinki.

### 2.2 Cell culture

#### 2.2.1 Human nasal epithelial cell culture

Human nasal tissues were obtained from patients with CRS during surgery. The procedure was approved by the Ethics Committee of Yonsei University College of Medicine. The tissues were treated with 0.1% protease (type XIV) in Dulbecco’s Modified Eagle Medium (DMEM)/F12 (Lonza) media with penicillin-streptomycin (pen-strep) overnight at 4°C. The scratched surface of the tissue and dissociated epithelial cells were washed three times. Human nasal epithelial cells were cultured as previously described (24). Suspended epithelial cells were seeded at 3 × 10^4^ cells per dish into 10-cm plastic tissue culture dishes with HNEC Subculture medium (**Supplementary Table S2**). Serially passaged HNECs (10^5^ cells/culture, 2 × 10^4^ cells/cm^2^) were seeded in 0.5 mL of culture medium onto the surface of Transwell culture inserts (Corning) with HNEC air-liquid interface (ALI) culture medium (**Supplementary Table S2**). On day 2, an ALI was established by eliminating the medium from the apical compartment and providing media to the cultures solely through the basal compartment. The ALI culture medium was changed every 2 days with the addition of retinoic acid (50nM). Cytokines (IL-4, IL-13, IL-1β, TNF-α, and IFN-γ; 5 ng/mL each) were added to the basal medium beneath the Transwell inserts.

#### 2.2.2 Human nasal fibroblast culture

After separating the nasal epithelial cell nasal polyps, the tissue was washed twice with phosphate-buffered saline (PBS) and then chopped into small pieces in a 10-cm dish before adding 10 mL of fibroblast culture medium (**Supplementary Table S2**). Cells were subcultured before reaching 80% confluence, and experimental treatments were performed at passage 2 using cells seeded into 12-well plates. Before treatment, the cells were starved overnight in fibroblast serum-free medium (**Supplementary Table S2**). At the initial stage of culture establishment, immunostaining for vimentin was performed to verify fibroblast identity, and α-SMA staining was used to assess the activation status and determine a suitable passage range for experimental use. Cytokines (IL-4, IL-13, IL-1β, TNF-α, and IFN-γ; 5 ng/mL each) were added directly to the culture medium covering the HNFs.

#### 2.2.3 Human nasal cell co-culture

For the epithelial cell and fibroblast co-culture model, HNECs were cultured on Transwell inserts at the ALI for 14 days to achieve differentiation and maturation. Simultaneously, HNFs were seeded at 10^4^ cells per well on the basolateral surface of a 12-well plate with the same dimensions as the Transwell plate. Following initial seeding, the HNFs were starved overnight. After starvation, Transwell inserts containing HNECs were placed into the Transwell plate above the HNFs. IL-4 (5 ng/mL) was added to the basal medium beneath the Transwell inserts to stimulate both cell types during co-culture.

#### 2.2.4 MG63 cell culture

MG63 cells were subcultured in 75 cm2 flasks with MG63 culture medium (**Supplementary Table S2**). Serially passaged MG63 cells were seeded in 1 mL of MG 63 osteogenic medium (**Supplementary Table S2**) in 12-well plates.

#### 2.2.5 Conditioned medium

Human nasal fibroblasts (HNFs) were treated with IL-4 (5 ng/mL) for 2 days, after which the culture supernatant was collected. To selectively retain periostin while removing IL-4 and other low-molecular-weight cytokines, the conditioned medium was concentrated using Amicon Ultra centrifugal filter units with a 30 kDa molecular weight cutoff (Millipore). This filtering step effectively excluded IL-4 (∼15-20 kDa) and retained the secreted periostin. The concentrated medium was referred to as CM-HNF. The concentration of periostin in the secretion (HNF) was quantified using ELISA (R&D Systems, DY3548B). To ensure consistency across experimental conditions, recombinant human periostin (rhPOSTN) was diluted to 200 ng/mL, and CM-HNF was adjusted to the same concentration based on the ELISA measurements. To neutralize the secreted periostin, CM-HNF was pretreated with a periostin-neutralizing antibody (R&D Systems, AF3548) at a final concentration of 10 µg/mL, sufficient to 200 ng/mL of periostin. The antibody was added directly to CM-HNF and incubated for 1 h at 37°C prior to application to cells.

### 2.3 ECRS model

#### 2.3.1 Animals

Six-week-old female C57BL/6J mice were obtained from the Jackson Laboratory. Periostin knockout (KO) mice (B6;129-*Postn^tm1Jmol^*/J) were provided by Prof. Sang-Wook Kim of Gyeongsang National University Hospital. The Ethics Committee and Institutional Animal Care and Use Committee of the Yonsei University Health System approved the use of animals in this study (IACUC No. 2024-0048), which was performed in accordance with the ARRIVE guidelines.

#### 2.3.2 ECRS mouse model

In both the wild-type (WT) and KO ECRS groups, mice received intranasal doses of various airborne allergens three times a week for 12 weeks. In contrast, the corresponding WT and KO PBS groups were treated with PBS. The “multiple allergens” formulation consisted of a combination of allergen extracts and proteins dissolved in sterile PBS, totaling 30 μL. This mixture contained house dust mite extract (20 µg), *Aspergillus fumigatus* (20 µg), *Alternaria alternata* (20 µg), and *Staphylococcus aureus* protease (1 µg). The solution was administered intranasally, with 15 μL applied to each of the nostrils. This combination of clinically relevant aeroallergens was selected to recapitulate the complex antigenic exposure characteristic of human ECRS. This model has been shown to reliably induce Th2-type eosinophilic inflammation in the sinonasal mucosa. The mice were analyzed after 12 weeks (n = 6/group) based on methods described in a previous study (25). All mice were sacrificed 24 h after the final intranasal administration. Six mice were used in each group. Four mice were allocated for histological analysis, which was the primary focus of this study owing to its sensitivity to tissue-level variations. In addition, to assess periostin expression at the molecular level, one mouse per group was used for RNA extraction and another for protein analyses. These assays were performed as supportive assays and were not intended for statistical comparison. Mice were randomly assigned to treatment groups before intranasal administration. Although blinding during treatment and outcome assessment was not feasible owing to experimental constraints, all tissue processing, measurement sites, and analysis parameters were standardized and uniformly applied across all groups to minimize potential bias. Detailed information on the reagents used for ECRS modeling is provided in **Supplementary Table S3**.

### 2.4 Immunostaining

#### 2.4.1 Immunohistochemistry

Formalin-fixed paraffin sections (4 μm) were dewaxed in xylene, rehydrated in ethanol baths, and subjected to antigen retrieval using a microwave in 0.01 mol/L sodium citrate buffer (pH 6.0). Endogenous peroxidase activity was quenched with 3% methanolic hydrogen peroxide for 10 min at room temperature (20–25°C). Non-specific binding was blocked with 10% normal serum (VECTASTAIN Elite ABC Kit; Vector Laboratories, Newark, CA, USA) for 30 min at RT. Primary antibodies against periostin (ab14041) or RUNX2 (ab192286; both 1:100, Abcam) were applied at 4°C for 24 h. After washing with Tris-buffered saline, the slides were incubated with horseradish peroxidase-conjugated species-specific secondary antibodies (1:5000) for 30 min at RT. Signal amplification was performed using the indirect immunoperoxidase technique (DAKO Envision kit; Agilent Technologies Inc., Santa Clara, CA). Tissue sections were counterstained with Gill’s hematoxylin, dehydrated, and mounted using the DPX (Sigma).

#### 2.4.2 Immunofluorescence

Formalin-fixed paraffin sections (4 μm) were dewaxed in xylene and rehydrated using a graded series of ethanol (100%, 95%, 80%, and 70%). Antigen retrieval was performed using a steamer with IHC-Tek Epitope Retrieval Solution (IHC World). Sections were treated with 3% methanolic hydrogen peroxide for 10 min at room temperature (20–25°C), followed by a 5-min wash with TBST. Blocking was performed using 5% bovine serum albumin for 1 h. The sections were then incubated overnight at 4°C with primary antibodies against fibronectin (Abcam, ab2413), periostin (R&D Systems, AF2955), osteopontin (Abcam, ab218237), and α-SMA (Abcam, ab5694; all 1:200). After washing with TBST thrice for 5 min each, the sections were incubated with a secondary antibody (1:1000, Alexa Fluor IgG(H+L), Invitrogen). Cells were stained with DAPI (1:1000) for 10 min. Coverslips were mounted using VECTASHIELD Mounting Medium (Vector Laboratories) before analyzing the slides using a confocal laser-scanning microscope (LSM700 & 970; Zeiss). Detailed information on the antibodies used for immunostaining is provided in **Supplementary Table S3**.

### 2.5 Enzyme-linked immunosorbent assay

We performed ELISA to determine the periostin concentration using antibodies contained in the kit (R&D Systems, DY3548B) according to the manufacturer’s instructions. For ELISA analysis, human tissue samples were homogenized and lysed using RIPA buffer, followed by protein quantification using the BCA assay (Thermo Fisher Scientific) to ensure equal amounts of total protein across samples. Conditioned media from the cell culture experiments were collected directly without further processing. For Transwell-based cultures of HNECs, apical and basal compartments were collected separately: 200 µL of PBS was gently applied to the apical surface of HNECs and carefully collected using light pipetting without disturbing the cell layer, whereas the basal media from both HNECs and HNFs were harvested directly without additional manipulation. Equal protein quantities were used for ELISA to enable a direct comparison of periostin levels between the experimental conditions.

### 2.6 Western blotting

#### 2.6.1 Cell lysate preparation

The cell culture dish was washed with PBS before adding RIPA buffer containing a protein inhibitor cocktail. The adherent cells were scraped off the dish, transferred into a tube, and placed on ice for 40 min. The cell lysate mixture was centrifuged at 12,000 rpm at 4°C for 15 min, and the supernatant (lysate) was transferred to a fresh tube on ice.

#### 2.6.2 Blotting procedure

The protein concentration of the cell lysates was assessed using a bicinchoninic acid assay. For western blots, we used primary antibodies against fibronectin (Abcam, ab2413), periostin (Abcam, ab14041), C-periostin (Abcam, ab152099), osteopontin (Abcam, ab218237), α-smooth muscle actin (α-SMA; Abcam, ab5694), and β-actin (Santacruz, sc-47778). The secondary antibodies were goat anti-mouse IgG(H+L)-HRP (GenDEPOT, SA001-500) and goat anti-rabbit IgG(H+L)-HRP (GenDEPOT, SA002-500). Detailed information on the antibodies used for immunostaining is provided in **Supplementary Table S3**.

### 2.7 Real-time quantitative reverse transcription-polymerase chain reaction

Total RNA was isolated using TRIzol reagent (Ambion) according to the manufacturer’s protocol. Total RNA (1 µg) was reverse-transcribed using M-MLV Reverse Transcriptase (Invitrogen). Direct sequencing of the extracted DNA was performed using an Applied Biosystems 3730xl DNA analyzer (Thermo Fisher). qPCR was performed using a 7300 Real-Time PCR System (Applied Biosystems) and KAPA SYBR^®^ FAST qPCR Kit (Kapa Biosystems, Wilmington, MA, USA) following the manufacturer’s instructions. The qPCR primers used are listed in **Supplementary Table S4**.

### 2.8 Histopathology

Mouse heads were fixed in 4% paraformaldehyde overnight, decalcified in 10% ethylenediaminetetraacetic acid for 2 weeks, and embedded in paraffin. The tissues were cut into 4-μm sections. Histological changes in the nasal mucosa were analyzed using hematoxylin and eosin staining for inflammation, Sirius Red staining for eosinophil infiltration and polyp-like lesions, periodic acid-Schiff staining for goblet cell hyperplasia, and Masson’s trichrome staining for collagen deposition and thickness of epithelial and subepithelial layers. The measurement sites were consistent for all mice. Eosinophil aggregates were defined as clusters of eosinophils (> 20/high-power field [HPF]) in the sinonasal mucosa. Eosinophils and goblet cells were counted in nine HPFs (400× magnification) and summed for comparison purposes. The maximum mucosal thickness at the olfactory-respiratory epithelium transition zone was measured by averaging values from four areas for comparison. Collagen deposition was quantified as the percentage of collagen-stained region relative to the subepithelial area using ImageJ software (version 1.51j8; National Institutes of Health, Bethesda, MD, USA).

### 2.9 ALP staining

To assess osteogenic differentiation, alkaline phosphatase (ALP) staining was performed using the Alkaline Phosphatase Staining Kit (ab284936, Abcam) according to the manufacturer’s instructions. MG63 cells were seeded in 12-well plates and cultured under the specified conditions. After the desired treatment period, the cells were fixed with the fixative solution provided in the kit for 1–2 min at room temperature (20–25°C). Following fixation, the cells were incubated with ALP staining solution for 15–30 min in the dark. The stained cells were gently rinsed with distilled water and observed under a light microscope. The intensity and distribution of ALP-positive staining were analyzed to evaluate osteogenic activity.

### 2.10 Statistical analysis

Statistical analyses were performed using GraphPad Prism v.10. All quantitative data are expressed as the mean ± standard error of the mean (SEM). All *in vitro* experiments were independently repeated at least thrice using biological replicates. For the *in vivo* experiments, six mice per group were used. Among them, four mice were allocated for histological analyses, and the remaining two were used for protein and RNA extraction. The exact sample sizes (n) used for each assay are indicated in the corresponding figure legend. Information on the statistical tests used for each experiment is provided in the respective figure legends and Results section. Statistical significance was set at P < 0.05.

## 3 Results

### 3.1 Periostin expression correlates with the osteitis score of patients with CRS

**Figure 1:**
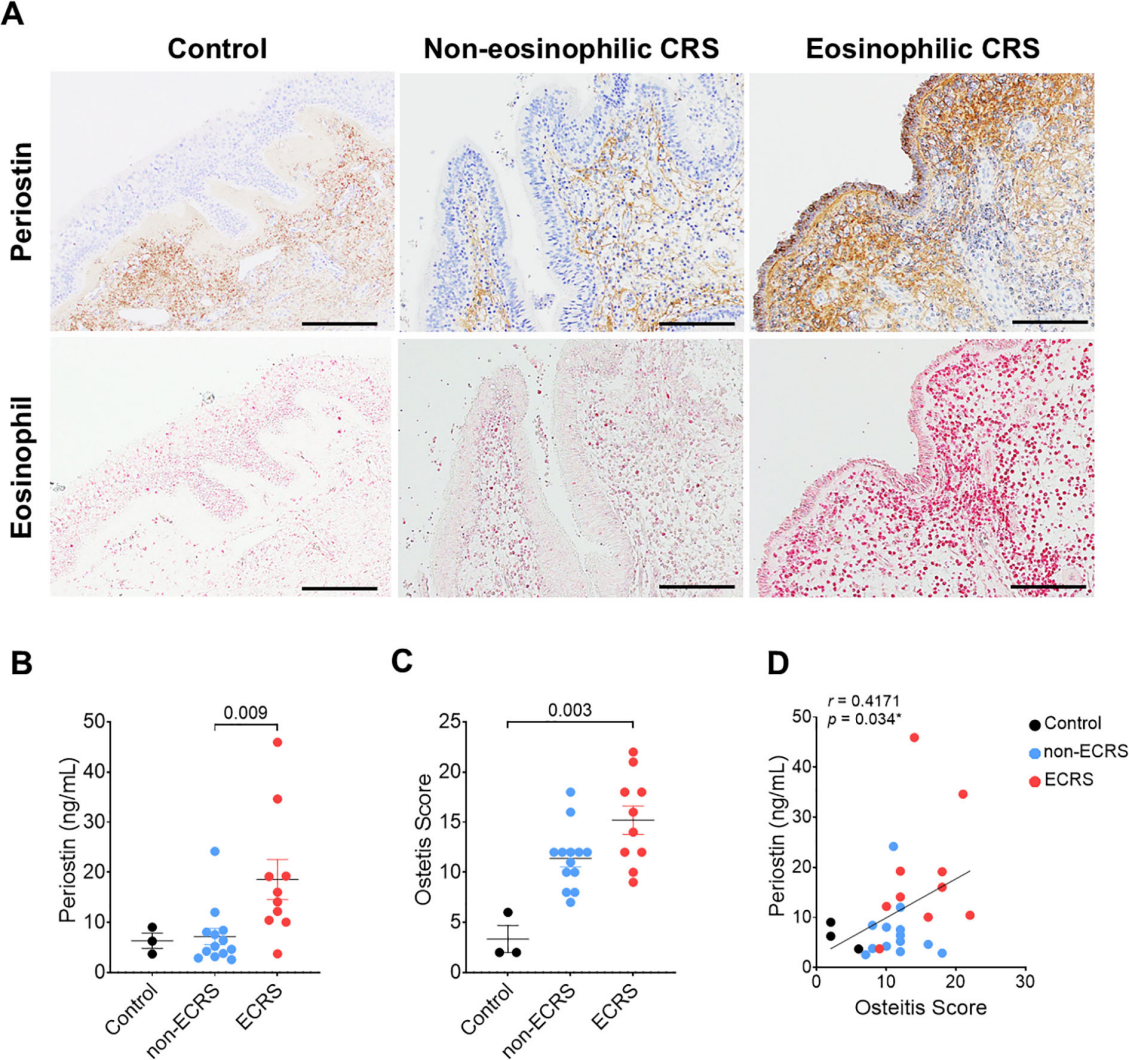
Correlation between periostin expression and osteitis severity in patients with CRS. Patients were categorized into non-ECRS and ECRS groups based on their JECSREC scores, with ECRS confirmed by eosinophil counts exceeding 70 cells per high-power field (HPF). **(A)** Representative photomicrographs of immunohistochemical staining for periostin (brown) and Sirius Red staining for eosinophil infiltration (pink) in the human nasal mucosa (scale bars = 100 μm). **(B)** Quantitative analysis of periostin levels in the nasal ethmoid mucosa, measured using ELISAs, in the control (n = 3), non-ECRS (n = 13), and ECRS groups (n = 10). **(C)** Osteitis severity was evaluated using the Global Osteitis Scoring Scale based on computed tomography images in the same group. **(D)** Correlation between periostin concentration and osteitis score in all groups. A moderate positive correlation was observed (r = 0.4171, *P* = 0.034; Spearman’s rank correlation). Results represent the mean ± SEM. Exact P values are shown in the graph. Statistical significance was assessed using the Kruskal–Wallis test for **(B)** and **(C)**. CRS, chronic rhinosinusitis; ELISA, enzyme-linked immunosorbent assay; ECRS, eosinophilic chronic rhinosinusitis; HPF, high-power field; JECSREC, Japanese Epidemiological Survey of Refractory Eosinophilic Chronic Rhinosinusitis; non-ECRS, non-eosinophilic chronic rhinosinusitis; SEM, standard error of the mean.

Periostin expression and osteitic changes were assessed in patients with ECRS, non-ECRS, and healthy controls. Classification was based on the Japanese Epidemiological Survey of Refractory Eosinophilic Chronic Rhinosinusitis score, which defined ECRS as >70 eosinophils per high-power field (HPF) after Sirius Red staining (**Figure 1A**). ELISA demonstrated significantly elevated periostin levels in the ECRS group compared to those in the non-ECRS group (*P =* 0.009, Kruskal–Wallis test) (**Figure 1B**). As the control group included only three samples (n = 3), non-parametric tests were applied to ensure robust group comparisons. Osteitis severity was evaluated using the Global Osteitis Scoring Scale (GOSS) on CT images. The ECRS group exhibited significantly higher osteitis scores than healthy control (*P =* 0.003, Kruskal–Wallis test) (**Figure 1C**). A moderate but statistically significant positive correlation was observed between periostin concentrations and osteitis scores (*r* = 0.4171, *P =* 0.034; Spearman’s rank correlation) (**Figure 1D**). Although this association suggests a potential link between periostin expression and osteitic remodeling.

### 3.2 Th2 cytokines induce periostin expression in HNECs and HNFs

**Figure 2:**
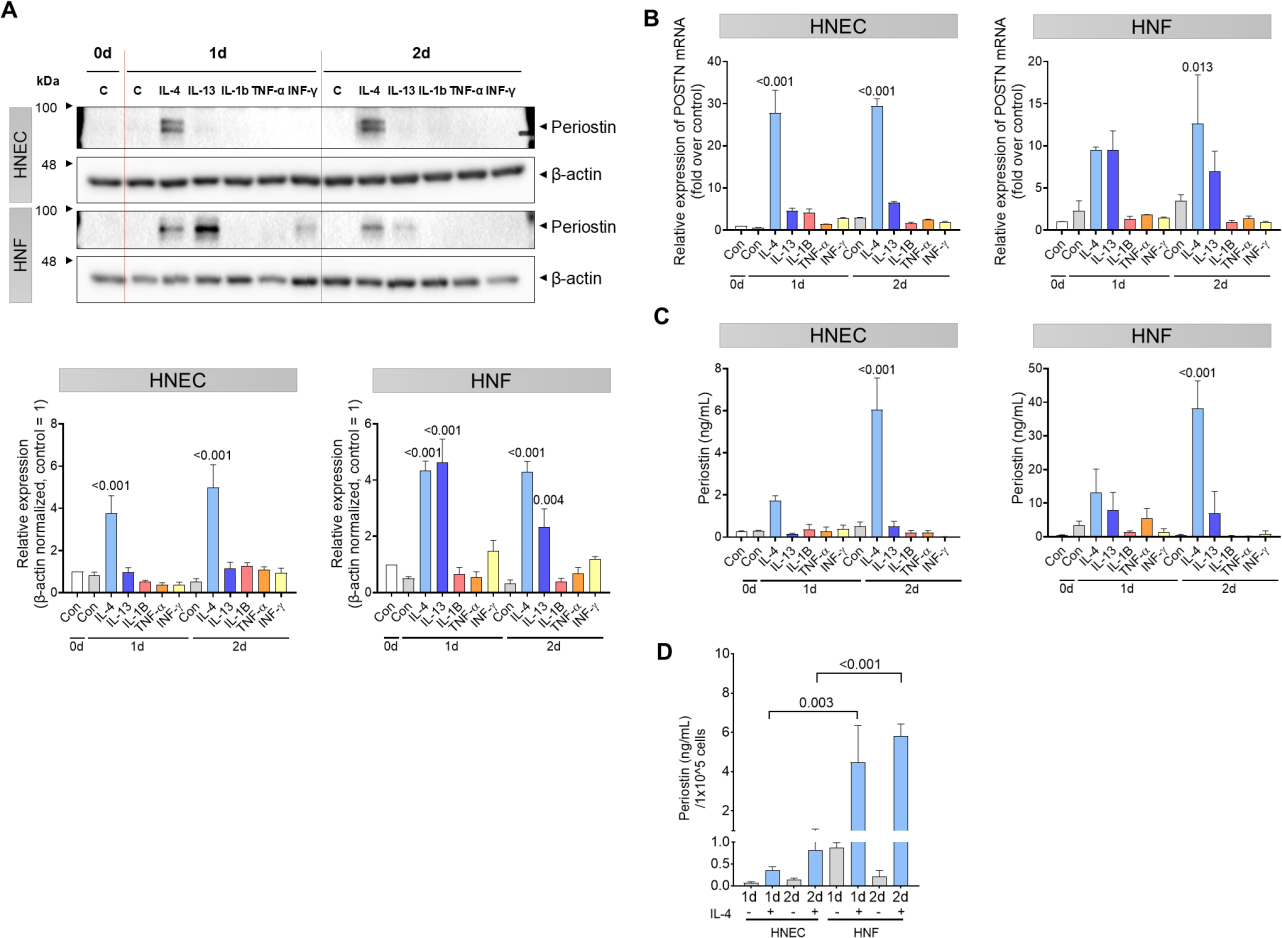
Th2 cytokine-induced periostin expression in HNECs and HNFs. HNECs and HNFs were treated with 5 ng/mL of IL-4, IL-13, IL-1β, TNF-α, and IFN-α and subsequently harvested on days 0, 1, and 2. **(A)** Western blot analysis of periostin expression levels in HNECs and HNFs treated with each cytokine for 0–2 days. β-actin used as a loading control for normalization. Band intensities were quantified from three independent experiments, normalized to the control group (set as 1), and expressed as relative intensities. **(B)** Quantitative RT-PCR analysis of *POSTN* mRNA levels in HNECs and HNFs following cytokine treatment, with normalized expression values on day 0. **(C)** Measurement of periostin concentrations in the culture supernatants by ELISA. **(D)** Comparative analysis of periostin secretion per equivalent number of cells quantified by ELISA. All experiments were independently repeated three times. Results represent the mean ± SEM. Exact P values are shown in the graph. Statistical analysis was performed using two-way ANOVA with Dunnett’s multiple comparisons test for **(A–C)** and Sidak’s multiple comparisons test for **(D)**. ELISA, enzyme-linked immunosorbent assay; HNEC, human nasal epithelial cell; HNF, human nasal fibroblast; IFN, interferon; IL, interleukin; *POSTN*, periostin; SEM, standard error of the mean; TNF, tumor necrosis factor.

Human nasal epithelial cells (HNECs) and fibroblasts (HNFs) derived from nasal polyps were used to investigate periostin induction in response to Th2 and other inflammatory cytokines. Cells were treated with 5 ng/mL IL-4, IL-13, IL-1β, TNF-α, or IFN-α and harvested on days 0, 1, and 2. In HNECs, periostin expression in cell lysates was significantly increased on days 1 and 2 following IL-4 treatment (*P <* 0.001). In HNFs, both IL-4 and IL-13 treatments led to a significant increase in periostin expression on days 1 (*P <* 0.001 for both) and 2 (*P <* 0.001 for IL-4; *P =* 0.004 for IL-13, two-way ANOVA with Dunnett’s multiple comparisons test) (**Figure 2A**). *POSTN* mRNA expression also increased in response to IL-4 in HNECs, and in HNFs, expression increased notably after 2 d of stimulation. Periostin levels increased on days 1 and 2 following IL-4 treatment in HNECs (*P <* 0.001). In HNFs, periostin expression showed an increasing trend following both IL-4 and IL-13 treatment, although only the IL-4 treated group reached statistical significance at day 2 (*P =* 0.013, two-way ANOVA with Dunnett’s multiple comparisons test) (**Figure 2B**). ELISA analysis of concentrated culture media confirmed that secreted periostin levels followed a similar pattern, increasing with IL-4 in HNECs and with IL-4 and IL-13 in HNFs. Among them, only day 2 following IL-4 treatment resulted in a statistically significant increase in periostin levels in both cell types (*P <*0.001, two-way ANOVA with Dunnett’s multiple comparisons test) (**Figure 2C**). Finally, the number of HNECs and HNFs was counted to determine the primary source of periostin production. To account for differences in cell numbers, periostin secretion was normalized to per cell. Under IL-4 treatment, HNFs secreted significantly more periostin per cell than HNECs on days 1 and 2 (*P =* 0.003 and *P <*0.001, respectively; two-way ANOVA with Sidak’s multiple comparisons test) (**Figure 2D**). To further investigate periostin isoform diversity in nasal cells following IL-4 treatment, we compared the molecular weights of endogenous and recombinant periostin using western blot analysis. Recombinant human periostin (rhPOSTN) appeared as a single band at approximately 100 kDa, consistent with full-length protein. In contrast, periostin derived from HNEC and HNF lysates presented multiple bands centered around 85 kDa. Furthermore, an antibody recognizing the C-terminal region of periostin detected a band only in the rhPOSTN lane but not in the cell lysates, providing additional evidence that endogenous periostin expressed by HNECs and HNFs predominantly lacks the C-terminal region. Notably, owing to the higher expression level of periostin in HNFs, the samples were diluted to match the expression level of HNECs, confirming that both cell types express periostin isoforms of similar size. These findings support the presence of distinct spliced forms of periostin in nasal cells under inflammatory conditions (**Supplementary Figure S1**).

### 3.3 Synergistic effects of HNEC and HNF co-culture on periostin secretion

**Figure 3:**
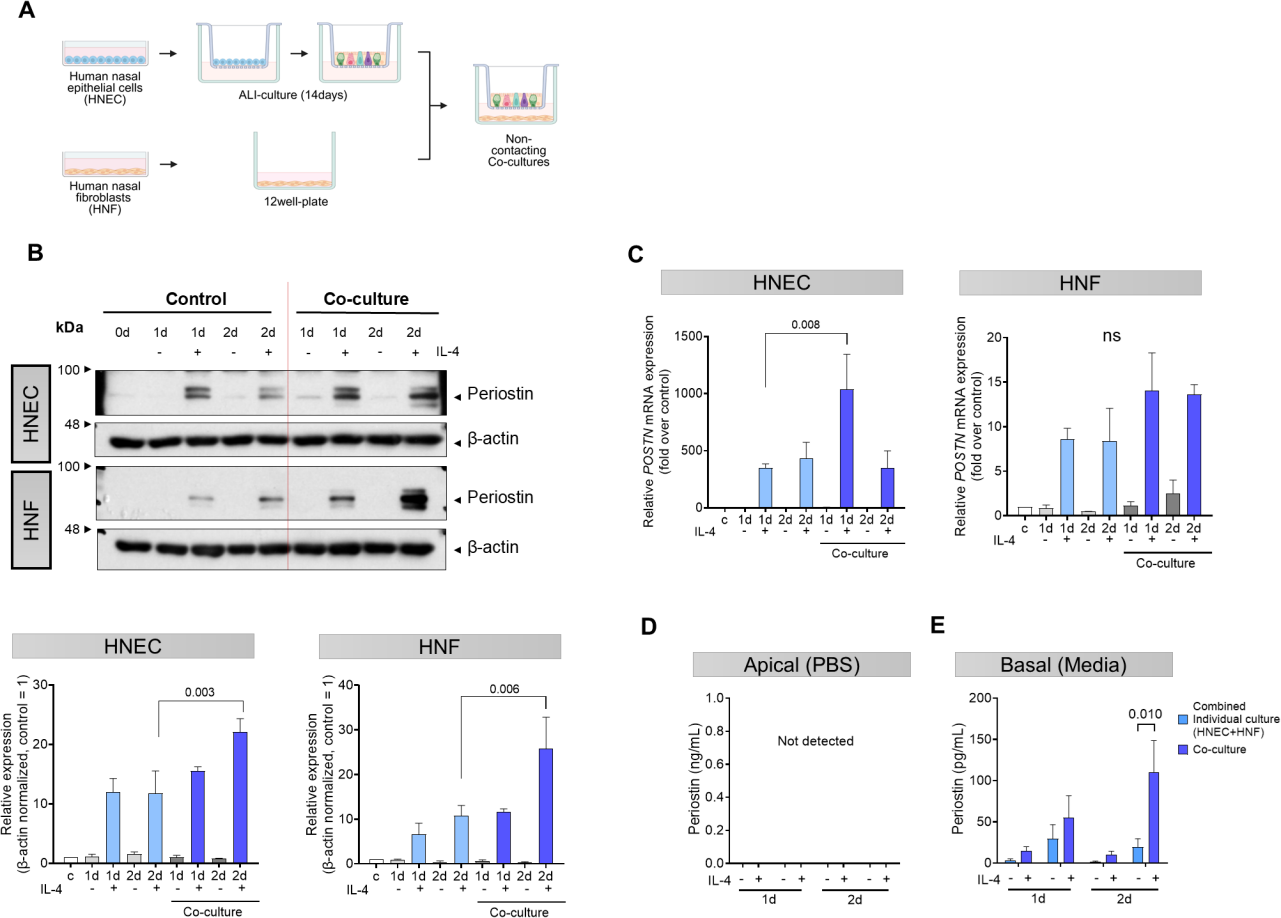
Synergistic effects of co-cultured HNECs and HNFs on periostin secretion. **(A)** Schematic representation of the non-contact co-culture system using Transwell inserts. After differentiation of HNECs by air-liquid interface (ALI) culture on Transwell inserts, the inserts were placed into plates containing HNFs in the bottom chamber. **(B)** Western blot analysis of periostin expression levels in separately cultured HNECs, separately cultured HNFs, and non-contact co-cultured HNECs and HNFs over a period of 0–2 days. β-actin was used as a loading control for normalization. Band intensities were quantified from three independent experiments, normalized to the control group (set as 1), and expressed as relative intensities. **(C)** Quantitative RT-PCR analysis of *POSTN* mRNA levels in HNECs and HNFs under different culture conditions, with values normalized to the level on day 0. **(D)** Comparative analysis of periostin levels in supernatants collected with PBS from the apical side of the Transwell in HNECs and co-cultured HNECs, quantified by ELISA. **(E)** Comparative analysis of periostin levels in supernatants collected from the basolateral side of the Transwell in HNECs, HNFs, and the co-culture condition, quantified by ELISA. All experiments were independently repeated three times. Results represent the mean ± SEM. Exact P values are shown in the graph. Statistical analysis was performed using two-way ANOVA with Sidak’s multiple comparisons test **(B–E)**. ALI, air-liquid interface; ELISA, enzyme-linked immunosorbent assay; HNEC, human nasal epithelial cell; HNF, human nasal fibroblast; SEM, standard error of the mean.

To investigate the interaction between HNECs and HNFs in periostin production, we conducted experiments using IL-4, a cytokine that elevates periostin levels in nasal cells, to determine whether both cell types influence each other. We used a Transwell system that allowed for non-contact co-culture by placing HNECs on Transwell inserts and HNFs in the bottom well of the plate, with IL-4 (5 ng/mL) added to the basal medium (**Figure 3A**). Periostin levels in the cell lysate were elevated in both cell types when co-cultured compared to the levels when the cell types were individually cultured. This synergistic increase was more pronounced in HNFs in the co-culture group. On day 2, under IL-4 stimulation, periostin expression was significantly higher in the co-culture group than in the monoculture group in both HNECs (*P =* 0.003) and HNFs (*P =* 0.006, two-way ANOVA with Sidak’s multiple comparisons test) (**Figure 3B**). In HNECs, *POSTN* mRNA levels were significantly increased in the co-culture setup with IL-4 treatment compared to IL-4 treated monocultures on day 1 (*P =* 0.008, two-way ANOVA with Sidak’s multiple comparisons test), indicating an enhanced response under co-culture conditions. In HNFs, although *POSTN* mRNA levels were higher in the co-culture + IL-4 condition than in the monoculture, this difference was not statistically significant (**Figure 3C**). Periostin secretion was quantified in both the apical and basal compartments. Periostin was not detected in the apical wash, but was clearly present in the basal medium, indicating basolateral secretion (**Figures 3D, E**). An analysis of the media revealed that the amount of periostin secreted was higher in the co-culture group than in the combined total from individually cultured HNECs and HNFs. This overall increase was most prominent under IL-4 stimulation, showing a statistically significant elevation only in the day 2 IL-4 treated group (*P =* 0.010, two-way ANOVA with Sidak’s multiple comparisons test) (**Figure 3E**).

### 3.4 Secreted periostin from HNFs mediates MG63 osteogenesis

**Figure 4:**
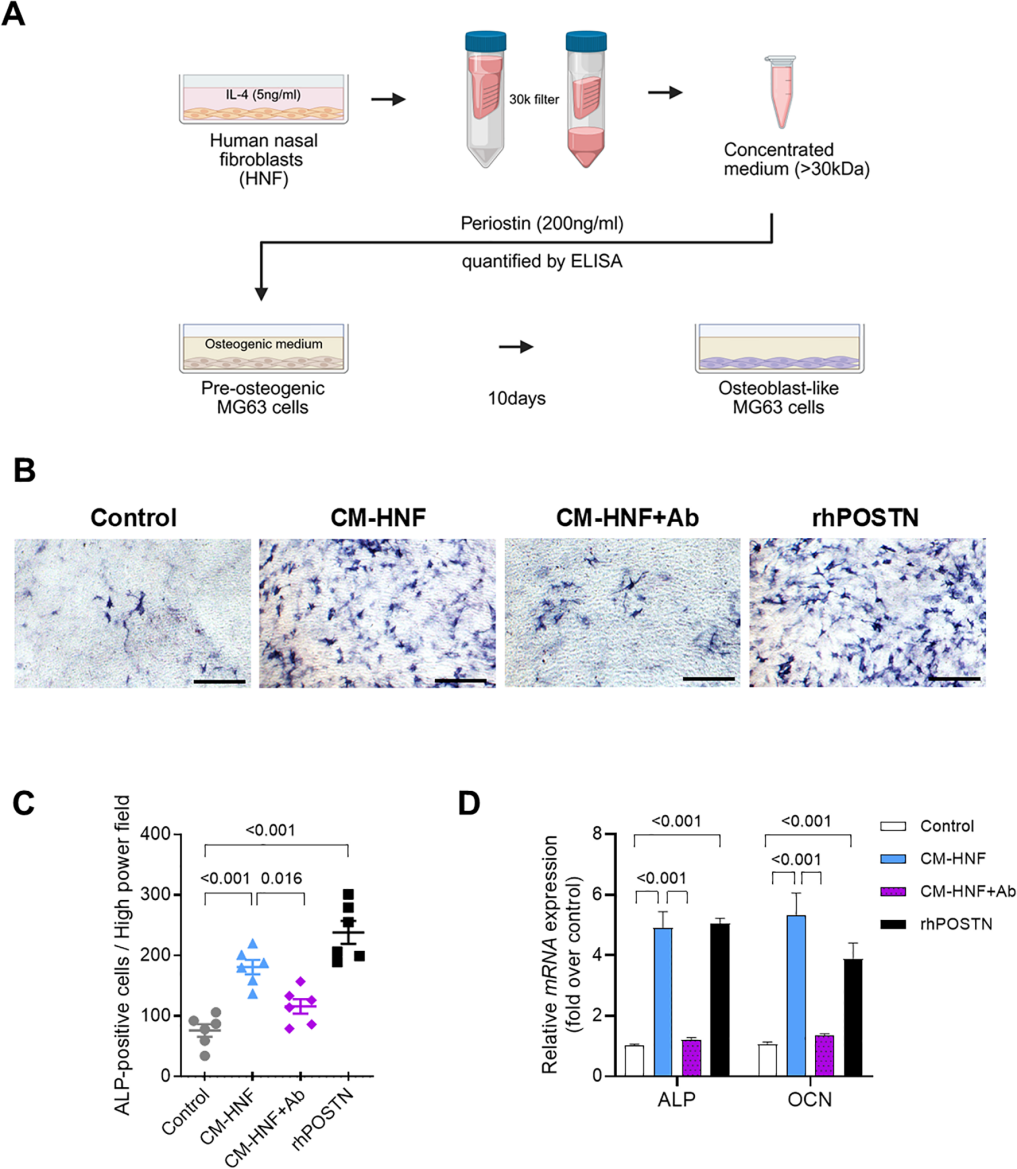
Periostin secreted by IL-4 treated nasal fibroblasts mediates the osteogenesis of MG63 cells. **(A)** Schematic overview of the conditioned medium preparation and MG63 osteogenic differentiation assay. HNFs were stimulated with IL-4 (5 ng/mL) for 2 days, and the conditioned medium was concentrated using a 30 kDa filter. Periostin concentrations were quantified by ELISA, and the periostin-containing medium was designated as CM-HNF. MG63 cells were cultured in osteogenic medium supplemented with CM-HNF, CM-HNF pretreated with a periostin-neutralizing antibody (CM-HNF + Ab), or recombinant human periostin (rhPOSTN, 200 ng/mL) for 10 days to induce osteogenic differentiation. **(B)** Representative photomicrographs of ALP staining showing osteoblast differentiation of MG63 cells induced by the osteogenic medium (scale bars = 500 μm). **(C)** Quantification of ALP-positive cells by counting the number of cells per high-power field in each well (n = 6, pooled from two independent experiments with three wells each). Statistical analysis was performed using one-way ANOVA with Tukey’s multiple comparisons test. **(D)** Quantitative RT-PCR analysis of ALP and OCN mRNA expression in MG63 cells under each treatment condition, with values normalized to those of the control group. Experiments were independently repeated three times. Statistical analysis was performed using two-way ANOVA with Tukey’s multiple comparisons test. Results represent the mean ± SEM. Exact P values are shown in the graph. Ab, antibody; ALP, alkaline phosphatase; rhPOSTN, recombinant human periostin; ELISA, enzyme-linked immunosorbent assay; HNF, human nasal fibroblast; IL, interleukin; OCN, osteocalcin; SEM, standard error of the mean.

To explore the influence of periostin on bone formation, we collected conditioned media from nasal fibroblasts treated with IL-4 for 2 days. A 30 kDa filter was used to concentrate the conditioned medium, allowing the retention of periostin while removing IL-4 and other molecules with sizes < 30 kDa (**Figure 4A**). A western blot analysis confirmed that the secreted periostin was > 63 kDa (**Figure 2A**). Differentiation of MG63 cells into osteoblasts was induced over 10 days using MG63 osteogenic medium. MG63 cells were treated with conditioned medium from IL-4 treated human nasal fibroblasts (CM-HNF), CM-HNF pretreated with periostin-neutralizing antibodies (CM-HNF + Ab), or recombinant human periostin (rhPOSTN). The concentration of periostin in the medium was confirmed using ELISA. After 10 days, the number of alkaline phosphatase (ALP)-positive cells was significantly increased in the CM-HNF and rhPOSTN groups than in the control group (*P <* 0.001 for both), indicating enhanced osteogenic activity. In contrast, the group treated with CM-HNF+Ab exhibited a reduction in the number of ALP-positive cells (*P =* 0.016, one-way ANOVA with Tukey’s multiple comparisons test), demonstrating that the osteogenic effect was largely mediated by periostin (**Figures 4B, C**). We also examined the mRNA expression levels of *ALP* and osteocalcin (*OCN*), which are key markers of osteogenic differentiation. The results showed that both mRNA levels were significantly elevated in the CM-HNF and rhPOSTN groups than in the control group (*P <* 0.001 for both). This increase in mRNA levels corroborated the enhanced osteogenic activity observed after ALP staining. Conversely, the CM-HNF +Ab group showed a marked decrease in *ALP* and *OCN* mRNA levels (*P* < 0.001, Two-way Repeated Measures ANOVA with Sidak’s multiple comparisons test) (**Figure 4D**).

### 3.5 Effects of periostin deficiency on inflammation in a murine model of ECRS

**Figure 5:**
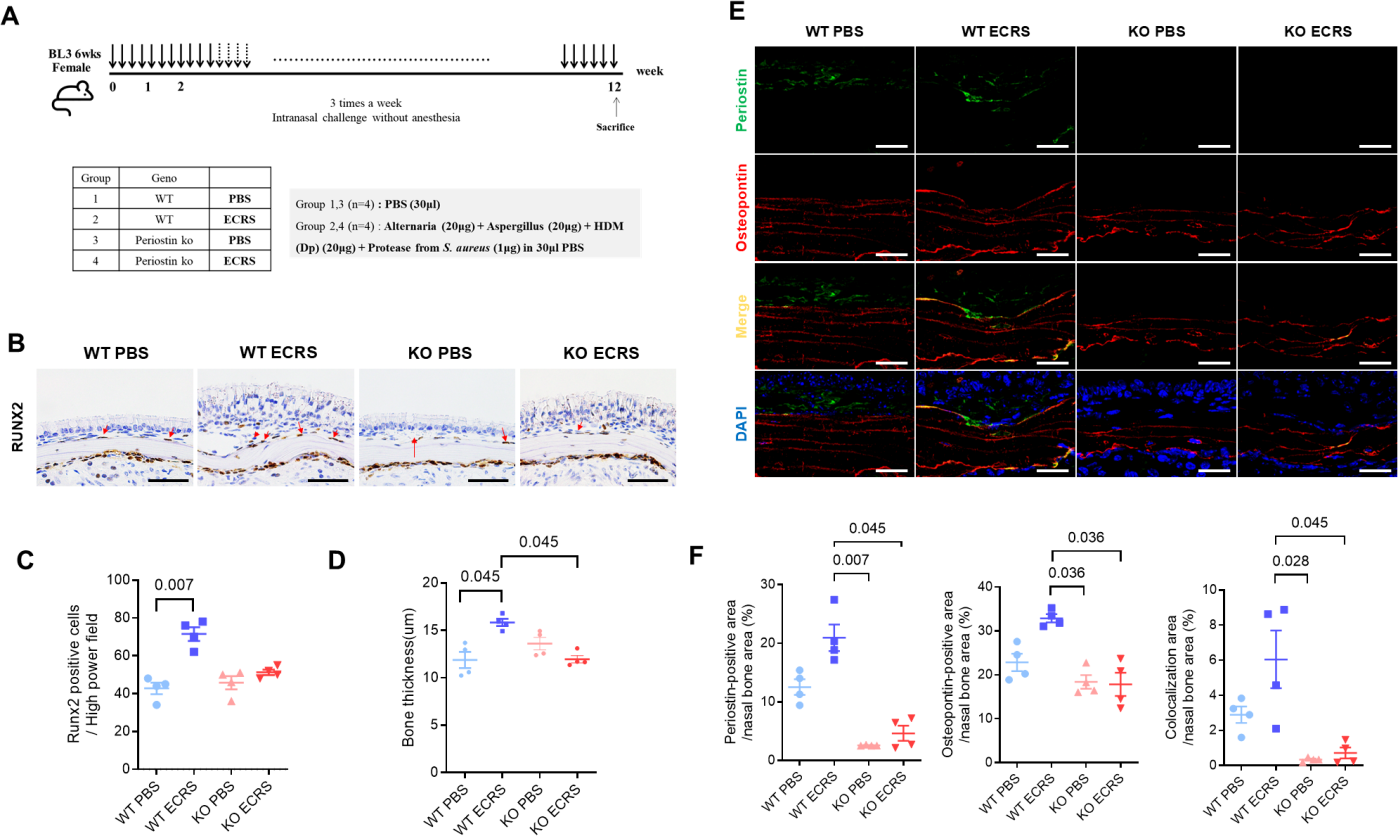
Effect of periostin deficiency on osteogenesis in a mouse model of ECRS. **(A)** Protocols for the development of eosinophilic rhinosinusitis in WT and KO mice. The ECRS condition was induced by exposing the mice to multiple allergens that stimulate Th2-type inflammation, mimicking clinical ECRS. **(B)** Representative photomicrographs of immunohistochemical RUNX2 staining in the ventral meatus area of the nasal cavity, showing RUNX2-positive cells along the nasal bone outline (scale bars = 50 μm). **(C)** Quantification of RUNX2-positive cells in the subepithelium of the nasal bone, with cell counts performed from sections of the ventral meatus area (n = 4). **(D)** Measurement of bone thickness in the ventral meatus of the nasal cavity across groups. Bone thickness was measured at five different locations in each mouse (n = 4). **(E)** Representative immunofluorescence images of periostin (green), osteopontin (red), and nuclei (blue) in the ventral meatus area of the nasal cavity (scale bars = 50 μm). Colocalization of periostin and osteopontin appeared as yellow signals resulting from the overlay of green and red fluorescence. **(F)** Quantification of periostin and osteopontin-positive areas and their colocalization in the nasal bone. Immunofluorescence analysis was performed on nasal tissue sections (n = 4), with paired regions (left and right) sampled from the same anatomical site of the ventral meatus. The positive signal areas for periostin and osteopontin, as well as the areas colocalized with periostin, were quantified using ImageJ. All values were expressed as a percentage of the total nasal bone area. Results represent the mean ± SEM. Exact P values are shown in the graph. Statistical analysis was performed by Kruskal–Wallis test with Dunn’s multiple comparisons test **(C, D, F)**. ECRS, eosinophilic chronic rhinosinusitis; KO, periostin knockout; PBS, phosphate-buffered saline; RUNX2, RUNX family transcription factor 2; SEM, standard error or the mean; WT, wild-type.

Multiple allergens can induce Th2 inflammation in the nasal cavity and mimic ECRS by inducing goblet cell hyperplasia, polyp-like lesions, and blood eosinophilia (25). To confirm the role of periostin in inflammatory diseases, we compared periostin knockout (KO) and wild-type (WT) ECRS models (**Figure 5A**). Periostin expression in the nasal mucosa was observed in WT mice; the periostin levels in tissue lysates of the WT ECRS group were significantly higher than those of the WT PBS group, indicating an association between periostin upregulation and ECRS. As expected, periostin was not detected in the KO mice (**Supplementary Figure S2A**). In addition, *POSTN* mRNA levels were higher in the WT ECRS group than in the WT PBS group, whereas both the KO groups exhibited barely any expression. These results confirmed the KO efficiency (**Supplementary Figure S2B**). IHC results further supported these findings by revealing periostin expression in the subepithelial regions of the nasal mucosa of WT mice. This expression was the most evident in the ventral meatus of the nasal cavity, and the WT ECRS group exhibited higher periostin expression than the WT PBS group. In contrast, the KO group did not express periostin, consistent with the protein and qPCR data (**Supplementary Figure S2C**). Quantitative analysis of eosinophil infiltration across the nasal mucosa revealed an overall increase in eosinophil counts in both ECRS groups compared to the PBS controls. Statistically significant increases were observed in the total eosinophil count when comparing WT ECRS to KO PBS (*P =* 0.016), as well as in specific anatomical regions: the ventral meatus and septum in KO ECRS versus KO PBS (*P =* 0.04, 0.018, respectively), and the maxillary turbinate in WT ECRS versus WT PBS (*P =* 0.012, Kruskal–Wallis test with Dunn’s multiple comparisons test). These findings support enhanced eosinophilic inflammation under ECRS conditions, although not all comparisons reached statistical significance (**Supplementary Figures S3A, B**). Periodic acid-Schiff staining was used to assess goblet cell hyperplasia in the nasal mucosa, which is crucial for mucus secretion. Although the WT and KO ECRS groups exhibited an apparent increase in goblet cell numbers compared to both PBS groups, this difference did not reach statistical significance, indicating that goblet cell hyperplasia under ECRS conditions was not consistently altered by periostin deficiency (**Supplementary Figures S4A, B**). These results indicate that the ECRS model was successfully established, as evidenced by increased eosinophil infiltration and goblet cell hyperplasia in both the WT and KO ECRS groups than in the PBS control groups. Notably, no significant differences were observed between the WT and KO ECRS mice, indicating that periostin deficiency did not significantly alter the inflammatory features of the model.

### 3.6 Effects of periostin deficiency on osteitic changes in a murine model of ECRS

Moreover, IHC revealed runt-related transcription factor 2 (RUNX2)-positive cells along the outline of the nasal bone, particularly in regions contacting the subepithelium (**Figure 5B**). RUNX2 is a key transcription factor associated with osteogenic activity, and its presence indicates active bone formation. The number of RUNX2-positive cells was notably increased in the WT ECRS group compared to that in the WT PBS group (*P* = 0.007, Kruskal–Wallis test with Dunn’s multiple comparisons test). In contrast, the number of RUNX2-positive cells did not differ between the KO ECRS and KO PBS groups. Although the number of RUNX2-positive cells appeared lower in the KO ECRS group than in the WT ECRS group, this difference did not reach statistical significance (**Figure 5C**). Further supporting these findings, measurements of bone thickness in the ventral meatus of the nasal cavity revealed that the nasal bones in the WT ECRS group were significantly thicker than those in the KO ECRS group (*P =* 0.045). In addition, WT ECRS showed greater bone thickness than KO ECRS, with a similar level of statistical significance (*P =* 0.045, Kruskal–Wallis with Dunn’s multiple comparisons test) (**Figure 5D**). In addition, IF analysis showed that periostin (green) was expressed in the subepithelium of WT PBS and WT ECRS, whereas osteopontin (red) was broadly expressed throughout the nasal bone in all groups. The colocalization of periostin and osteopontin(yellow) was specifically observed along the nasal bone outline in WT ECRS (**Figure 5E**). Quantitative analysis of the IF images was performed by measuring the positive area within the nasal bone region, expressed as a percentage of the total area. The periostin-positive area was significantly greater in WT ECRS than in KO PBS (*P =* 0.007) and KO ECRS (*P =* 0.045) groups. For osteopontin, a significant difference was observed between the WT and KO ECRS groups (*P =* 0.036). In addition, the area of colocalization between periostin and osteopontin was significantly higher in WT ECRS than in KO ECRS (*P =* 0.045, Kruskal–Wallis with Dunn’s multiple comparisons test) (**Figure 5F**). western blot analysis confirmed that osteopontin levels in the nasal mucosa were increased only in the WT ECRS group, and the osteopontin levels did not differ between the KO ECRS and KO PBS groups (**Supplementary Figure S5**).

### 3.7 Effects of periostin deficiency on tissue remodeling in a murine model of ECRS

**Figure 6:**
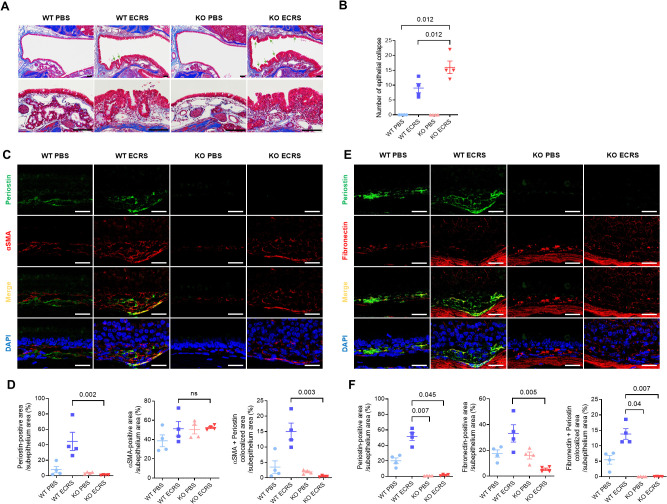
Effect of periostin deficiency on the maintenance of tissue structure in ECRS model mice. **(A)** Representative photomicrographs of Masson’s trichrome staining showing collagen (blue) deposition in the subepithelium of the nasal mucosa in different groups (scale bars = 50 μm). Representative photomicrographs showing epithelial collapse in the nasal cavity of the WT and KO ECRS groups, indicating a localized breakdown in the epithelial layer (scale bars = 50 μm). **(B)** Number of epithelial collapses in the nasal cavity. **(C)** Representative immunofluorescence images of periostin (green), α-SMA (red), and nuclei (blue) in the ventral meatus of the nasal cavity. Colocalization of periostin and α-SMA appeared as yellow signals resulting from the overlay of green and red fluorescence (scale bars = 50 μm). **(D)** Quantification of periostin, fibronectin-positive, and colocalization areas in the nasal subepithelium. **(E)** Representative immunofluorescence images of periostin (green), fibronectin (red), EMBP (white), and nuclei (blue) in the same anatomical region. **(F)** Quantification of periostin, fibronectin-positive, and colocalization areas in the nasal subepithelium. Immunofluorescence analysis was performed on nasal tissue sections (n = 4), with paired regions (left and right) sampled from the same anatomical site of the ventral meatus. The positive signal areas for periostin, α-SMA, and fibronectin, as well as the areas colocalized with periostin, were quantified using ImageJ **(D, F)**. All values were expressed as a percentage of the total nasal subepithelium. Results represent the mean ± SEM. Exact P values are shown in the graph. Statistical analysis was performed by Kruskal–Wallis test with Dunn’s multiple comparisons test **(B, D, F)**. α-SMA, alpha-smooth muscle actin; ECRS, eosinophilic chronic rhinosinusitis; KO, periostin knockout; PBS, phosphate-buffered saline; SEM, standard error or the mean; WT, wild-type.

Masson’s trichrome staining stained collagen in the nasal mucosa blue and distinguished the epithelium and subepithelium. The epithelial collapse region was evident in both the WT and KO ECRS groups (**Figure 6A**). This collapse demonstrated a localized breakdown of the epithelium in the respiratory area, possibly resulting from a weakened ECM structure. KO ECRS exhibited significantly more epithelial collapse than WT PBS and WT ECRS (P = 0.012 for both, Kruskal–Wallis with Dunn’s multiple comparisons test). Although the WT ECRS group showed a greater number of collapsed regions than the WT PBS group, this difference was not statistically significant (**Figure 6B**). To further characterize the histological changes associated with epithelial collapse, we assessed the epithelial and subepithelial thickness in the nasal cavity. Epithelial and subepithelial thicknesses were measured by averaging the values from three points within each area. In the epithelium, both WT ECRS and KO ECRS showed significant thickening in the ventral meatus compared to their respective PBS controls (*P =* 0.023 and *P =* 0.036, respectively). In the maxillary turbinate, epithelial thickness was also significantly increased in WT ECRS and KO ECRS than in KO PBS (*P =* 0.045 and *P =* 0.026, respectively; Kruskal–Wallis with Dunn’s multiple comparisons test). In contrast, the subepithelial thickness showed no statistically significant differences among the groups. Although both WT and KO ECRS tended to exhibit greater subepithelial thickening in the ventral meatus, particularly in KO ECRS, these differences were not statistically significant (**Supplementary Figure S6A**). To investigate the potential causes of epithelial collapse, we analyzed the expression of extracellular matrix (ECM) protein levels. First, the percentage of collagen-positive areas was measured to quantify collagen deposition in the subepithelial region. In Masson’s trichrome staining, collagen was visualized as blue, allowing a clear distinction between collagen-rich areas. The results showed increased collagen deposition in the subepithelium in both the WT and KO ECRS groups. However, collagen deposition did not differ between the two groups (**Supplementary Figure S6B**). To identify connections with ECM proteins, periostin (green) and α-SMA (red) expression were assessed using IF. Both markers were expressed in the subepithelium of all groups. Colocalization of periostin with α-SMA (yellow) was predominantly observed in the WT ECRS group. In this group, α-SMA and periostin were colocalized in fibroblasts exhibiting a stretched morphology (**Figure 6C**). Quantitative assessment was performed using IF images by defining the subepithelial area as the region of interest (ROI). Periostin expression was significantly higher in the WT ECRS group than in the KO ECRS group (*P* = 0.002). The α-SMA expression did not differ significantly among the groups. Colocalization analysis showed significantly greater colocalization between periostin and α-SMA in WT ECRS than in KO ECRS (P = 0.003), despite no difference in α-SMA expression alone, indicating that the difference arose primarily from periostin expression (**Figure 6D**). Similarly, immunofluorescence analysis of fibronectin (red) and periostin (green) showed overlapping signals in WT ECRS, particularly in the stretched fibroblasts (**Figure 6E**). Quantitative analysis revealed that periostin expression was significantly higher in WT ECRS than in KO ECRS (*P* = 0.045), and fibronectin expression was significantly increased in WT ECRS compared to KO ECRS (*P* = 0.005). Colocalization between fibronectin and periostin was also significantly higher in WT ECRS than in KO ECRS (*P* = 0.007, Kruskal–Wallis test with Dunn’s multiple comparisons test) (**Figure 6F**). α-SMA and fibronectin expression in the nasal mucosa was confirmed using western blotting. α-SMA expression did not differ between the groups; however, fibronectin levels were higher in the WT ECRS group than in the control group, based on the IF results (**Supplementary Figure S5**). To confirm the effect of secreted periostin on fibronectin expression, we treated HNFs with CM-HNF, which contains periostin, conditioned medium pretreated with periostin-neutralizing antibodies(CM-HNF+ Ab), and recombinant human periostin (rhPOSTN). Western blotting revealed increased fibronectin levels in CM-HNF than in CM-HNF+Ab (**Supplementary Figure S7**).

## 4 Discussion

ECRS patients with nasal polyps exhibit higher periostin and *POSTN* mRNA levels than those without nasal polyps, reflecting their involvement in Th2-driven inflammation (26). Our analysis of the nasal ethmoid mucosa from patients with CRS revealed periostin expression in both ECRS and non-ECRS patients, with more prominent subepithelial localization and markedly elevated levels in the ECRS group (**Figures 1A, B**). Radiologic evaluation using the Global Osteitis Scoring Scale demonstrated increased bone thickening in patients with CRS compared to healthy controls, with the ECRS group exhibiting significantly higher scores than the non-ECRS group (**Figure 1C**). Furthermore, periostin concentrations in the nasal tissue were moderately positively correlated with osteitis severity (**Figure 1D**). These findings support the hypothesis that periostin is not only a marker of eosinophilic inflammation but also contributes to osteogenic remodeling in the nasal mucosa. The observed association between periostin levels and osteitic changes underscores its potential role as a mechanistic link between Th2 inflammation and chronic bone pathology in patients with ECRS.

Periostin expression and release are regulated by Th2 cytokines in airway cells, including fibroblasts, epithelial cells, and endothelial cells (7–11, 27, 28). In our study, IL-4 induced a robust increase in periostin expression in both human nasal epithelial cells (HNECs) and fibroblasts (HNFs), whereas IL-13 upregulated periostin expression only in fibroblasts (**Figure 2A**). This differential responsiveness implies the existence of distinct regulatory pathways between cell types. *POSTN* mRNA expression increased over time with IL-4 treatment in both cell types, although the kinetics and magnitude differed (**Figure 2B**). Secreted periostin followed a similar pattern: IL-4 enhanced periostin release in both HNECs and HNFs, whereas IL-13 acted only on fibroblasts and was less effective, and periostin levels induced by IL-4 increased over time (**Figure 2C**). Moreover, HNFs released more periostin than HNECs per cell, making HNFs the primary source of periostin under Th2 cytokine stimulation (**Figure 2D**). These findings indicate that nasal fibroblasts, rather than epithelial cells, are the predominant periostin-producing cells in response to Th2 cytokines, providing a potential cellular target for therapeutic intervention. Within 2 days of IL-4 stimulation, periostin secretion progressively increased, indicating that Th2 cytokine exposure enhances periostin production in patients with ECRS. Although a progressive increase in periostin secretion was observed over the 2-day period, this time frame was limited by the use of serum-free media, which was necessary to avoid confounding protein components during the ELISA-based quantification of periostin. Thus, further investigation is required to determine whether periostin production is sustained or further amplified under prolonged Th2 cytokine stimulation, as would occur in chronic disease settings such as ECRS. In addition, Periostin derived from both HNECs and HNFs showed multiple bands centered around 85 kDa, and the absence of C-terminal detection in these cell lysates further supports that endogenously expressed periostin predominantly lacks the C-terminal domain. These findings indicate that nasal cells produce distinct periostin isoforms under inflammatory conditions, which may influence its functional properties in the extracellular environment (**Supplementary Figure S1**).

Non-contact co-culture of HNECs and HNFs demonstrated increased periostin levels in both cell lysates (**Figure 3B**), indicating that their interaction promotes periostin expression. The increase was greater in HNFs, indicating that co-culture conditions strongly influence periostin expression in fibroblasts. Co-culture significantly elevated *POSTN* mRNA levels in HNECs on day 1, indicating that HNFs promote periostin expression in epithelial cells. However, *POSTN* mRNA levels in HNFs did not vary between co-cultures and separate cultures (**Figure 3C**), implying that periostin regulation in fibroblasts occurs primarily at the protein level. Secreted periostin was detected only in the basolateral media, not in the apical media, indicating basal secretion by HNECs (**Figures 3D, E**). The co-culture media showed greater secretion than the combined media from separate cultures, indicating a synergistic effect on periostin release upon IL-4 stimulation (**Figure 3E**). This finding indicates a complex paracrine interaction between fibroblasts and epithelial cells in the nasal mucosa, reinforcing the hypothesis that periostin amplifies chronic inflammation in ECRS through cell-cell signaling.

IL-4 stimulation increased periostin secretion in nasal cells, predominantly in HNFs. Treatment of MG63 cells with conditioned medium from IL-4 stimulated HNFs (CM-HNF) resulted in a significant increase in ALP-positive cells and enhanced osteogenic activity, comparable to that observed with recombinant periostin. In contrast, this effect was markedly reduced in cells exposed to CM-HNF pretreated with periostin-neutralizing antibodies (CM-HNF + Ab), indicating that periostin played a central role in driving the observed osteogenic response (**Figures 4B, C**). Consistently, mRNA expression levels of ALP and osteocalcin (OCN), key markers of osteogenic differentiation, were significantly elevated in the CM-HNF and recombinant periostin groups but suppressed in the CM-HNF + Ab group (**Figure 4D**). These findings provide functional evidence that periostin derived from IL-4 activated fibroblasts can directly promote osteogenic differentiation *in vitro*. While these results support a role for periostin in Th2-associated bone remodeling, the physiological generalizability is limited by the use of MG63 cells, which are derived from osteosarcoma cell lines. Further validation using primary human osteoblasts will be necessary to confirm the relevance of these findings.

*In vivo* studies using murine models of ECRS showed periostin expression in the subepithelial nasal mucosa of WT mice, with increased protein and mRNA levels in WT ECRS mice; no periostin expression was found in KO mice (**Supplementary Figures S2A–C**). These findings confirm that periostin is upregulated in response to Th2-driven inflammation. Despite the absence of periostin in KO mice, eosinophil infiltration was similarly increased in both WT and KO ECRS groups than in PBS controls, indicating that periostin is not essential for eosinophil recruitment (**Supplementary Figure S3**). This indicates that while periostin may modulate aspects of eosinophilic inflammation, its presence is not required for eosinophil accumulation in the nasal mucosa. Goblet cell hyperplasia was also observed in both ECRS groups, regardless of periostin expression, further indicating that periostin does not play a critical role in mucus-producing epithelial changes under these conditions (**Supplementary Figure S4**). Overall, these results indicate that periostin deficiency does not significantly alter the key inflammatory features of ECRS in this model, including eosinophil infiltration and goblet cell expansion, and that its primary function may lie outside direct regulation of immune cell recruitment or mucus hypersecretion.

RUNX2-positive cells, indicative of osteogenic activity, were observed in WT ECRS mice but not in KO ECRS mice compared to their respective controls (**Figures 5B, C**). This difference indicates that periostin contributes to osteogenesis induction in inflamed nasal bones. In addition, the nasal bone was thicker in WT ECRS mice than in KO ECRS mice (**Figure 5D**). IF analysis revealed that osteopontin expression colocalized with periostin in the nasal bone, with notably increased signal intensity observed only in the WT ECRS group (**Figure 5E**). Osteopontin is an ECM protein involved in regulating both osteoblast and osteoclast activity, and its colocalization with periostin along the bone surface indicates a functional association. Quantitative analysis of the IF images demonstrated significantly higher osteopontin expression in WT ECRS mice than in KO ECRS mice (**Figure 5F**), whereas western blot analysis served as a qualitative confirmation using a representative sample (**Supplementary Figure S5**), reflecting the expression pattern observed in immunofluorescence analysis from a single animal. Furthermore, colocalization analysis revealed a significantly larger periostin–osteopontin overlapping area in WT ECRS, supporting a spatial association between the two proteins that may contribute to osteogenic changes under ECRS conditions. These findings indicate that periostin contributes to ECRS-associated osteitis by activating RUNX2 expression and enhancing osteopontin-mediated bone remodeling. In the context of chronic sinonasal inflammation, periostin may serve as a mediator linking inflammatory stimuli to pathological osteogenesis, thereby contributing to bone thickening observed in ECRS.

Periostin expression in the lower airway is primarily linked to fibrosis, collagen deposition, and epithelial-mesenchymal transition (9, 20). In the current model, increased epithelial collapse was observed in KO ECRS mice (**Figures 6A, B**). To investigate the underlying cause of this structural breakdown, we assessed the tissue remodeling features in both groups. Epithelial and subepithelial thickening similarly increased in both ECRS groups, indicating that periostin does not critically influence these gross morphological changes under chronic inflammatory conditions (**Supplementary Figure S6A**). Collagen deposition in the subepithelium was also comparable between WT and KO ECRS mice (**Supplementary Figure S6B**), and α-SMA expression did not differ between the groups in the IF (**Figure 6D**), indicating that periostin does not significantly affect general fibrotic remodeling or ECM contractility. In contrast, fibronectin expression was significantly higher in WT ECRS mice than in KO ECRS mice, based on IF analysis (**Figures 6E, F**). Given its colocalization with periostin in the subepithelium, these findings indicate a periostin-dependent regulatory mechanism for fibronectin in inflammatory conditions. Western blot analysis showed a similar trend, confirming the differential expression patterns observed using IF (**Supplementary Figure S5**), reflecting the expression pattern observed in immunofluorescence analysis from a single animal. Therefore, the disruption of epithelial integrity in KO ECRS mice may reflect weakened ECM support owing to reduced fibronectin content. This hypothesis was further supported by *in vitro* data showing that treatment with periostin-containing conditioned media increased fibronectin levels, whereas periostin-neutralizing antibodies suppressed this effect (**Supplementary Figure S7**). As the neutralizing antibody selectively blocked periostin in the conditioned medium, the observed reduction supports a direct extracellular role of secreted periostin in modulating fibronectin expression. These analyses were performed primarily to assess whether α-SMA and fibronectin expression levels were affected by secreted periostin, as the conditioned media contained either native or antibody-neutralized periostin. Notably, periostin expression in cell lysates remained elevated in the CM-HNF + Ab group, despite the use of neutralizing antibodies. This is likely because the antibody was applied only to the conditioned medium and did not affect the intracellular periostin levels. Thus, the periostin detected in the cell lysates may have been induced by other factors in CM-HNF, independent of neutralized extracellular periostin.

In conclusion, increased periostin levels play a dual role in Th2 inflammatory sinonasal disease. Periostin maintains tissue remodeling homeostasis during chronic inflammatory damage; however, periostin secreted in the nasal mucosa induces osteitis in sinonasal bone. Although this study compared wild-type and periostin-deficient mice under multi-allergen exposure, the inclusion of an anti-inflammatory treatment group would help to distinguish periostin-specific effects from broader inflammatory responses. Future studies incorporating these controls may provide clearer mechanistic insights. Although *in vivo* rescue experiments using recombinant periostin were not feasible owing to structural and delivery limitations, *in vitro* models using periostin knockdown or overexpression could provide a more tractable system to further explore its role in epithelial remodeling and matrix regulation.

## Data Availability

The datasets generated or analyzed during the study are available from the corresponding author on reasonable request.
