# Supplementary figures and images for: Inflammation-driven periostin in ECRS has contrasting effects on tissue structural integrity and osteitis
Source: Front Immunol. 2025 Jun 18;16:1596746. doi: 10.3389/fimmu.2025.1596746 (PMC12213678; doi:10.3389/fimmu.2025.1596746)

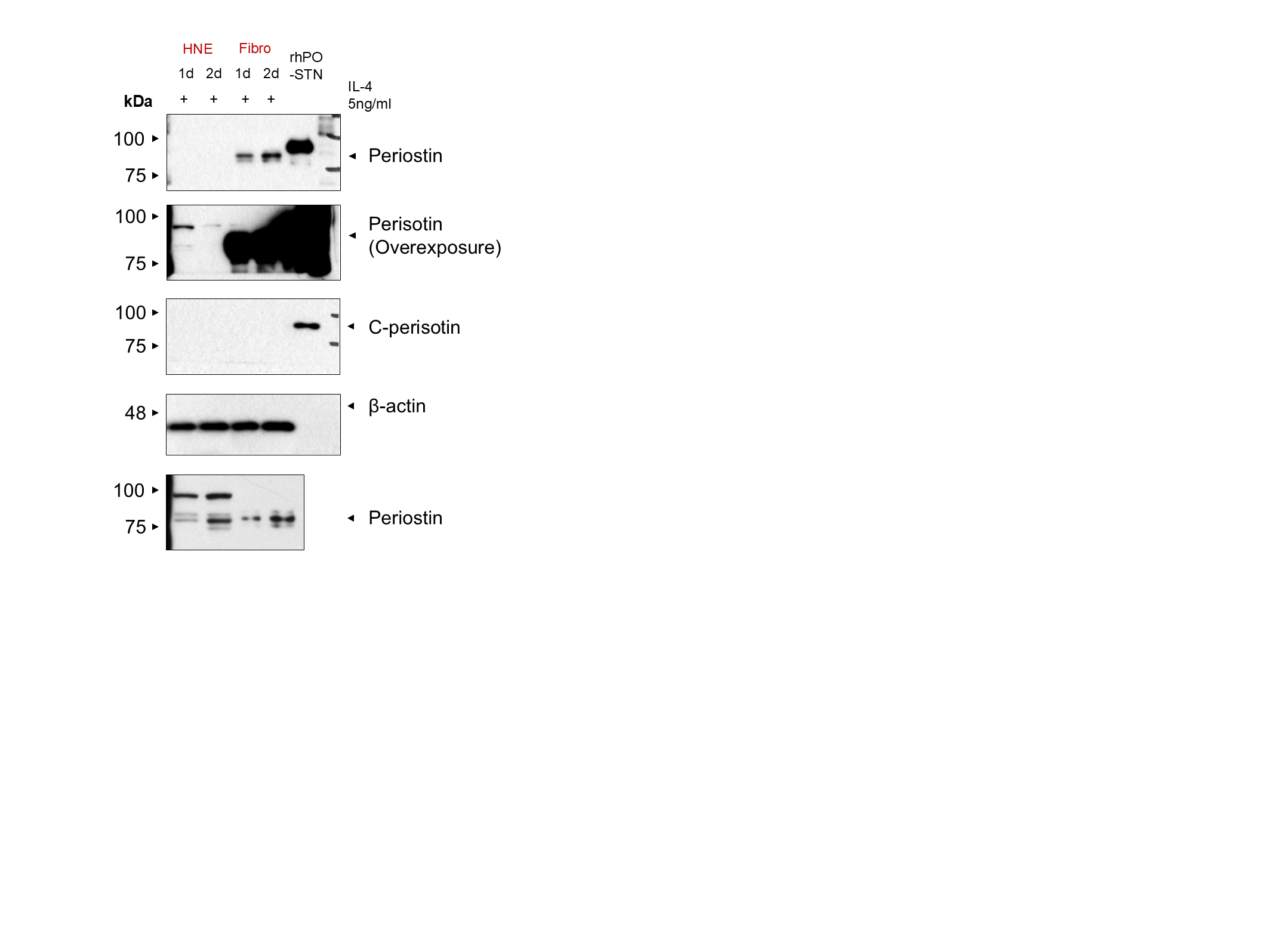

Supplement: Supplementary file 1 [file Image1.tif]

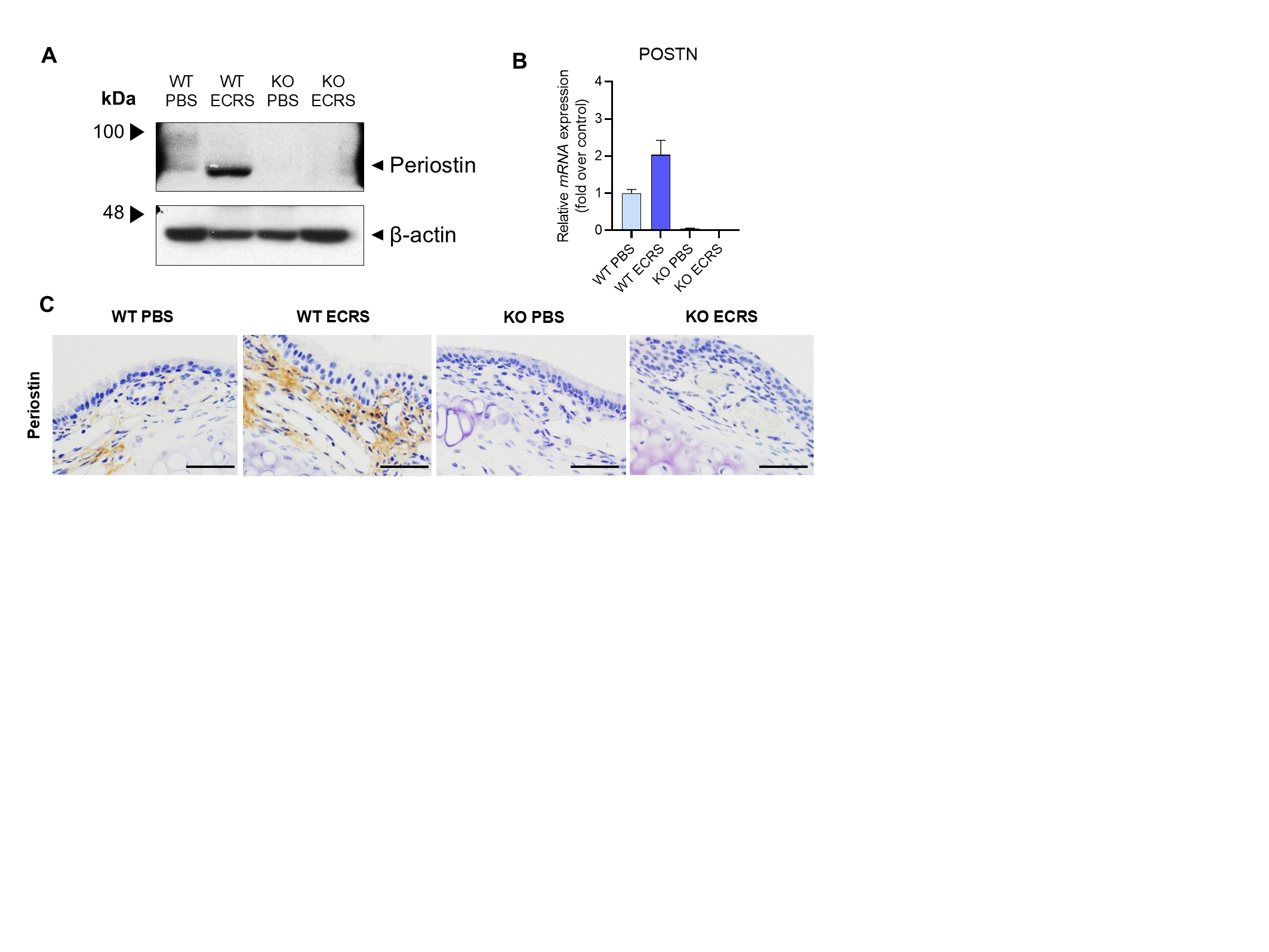

Supplement: Supplementary file 2 [file Image2.tif]

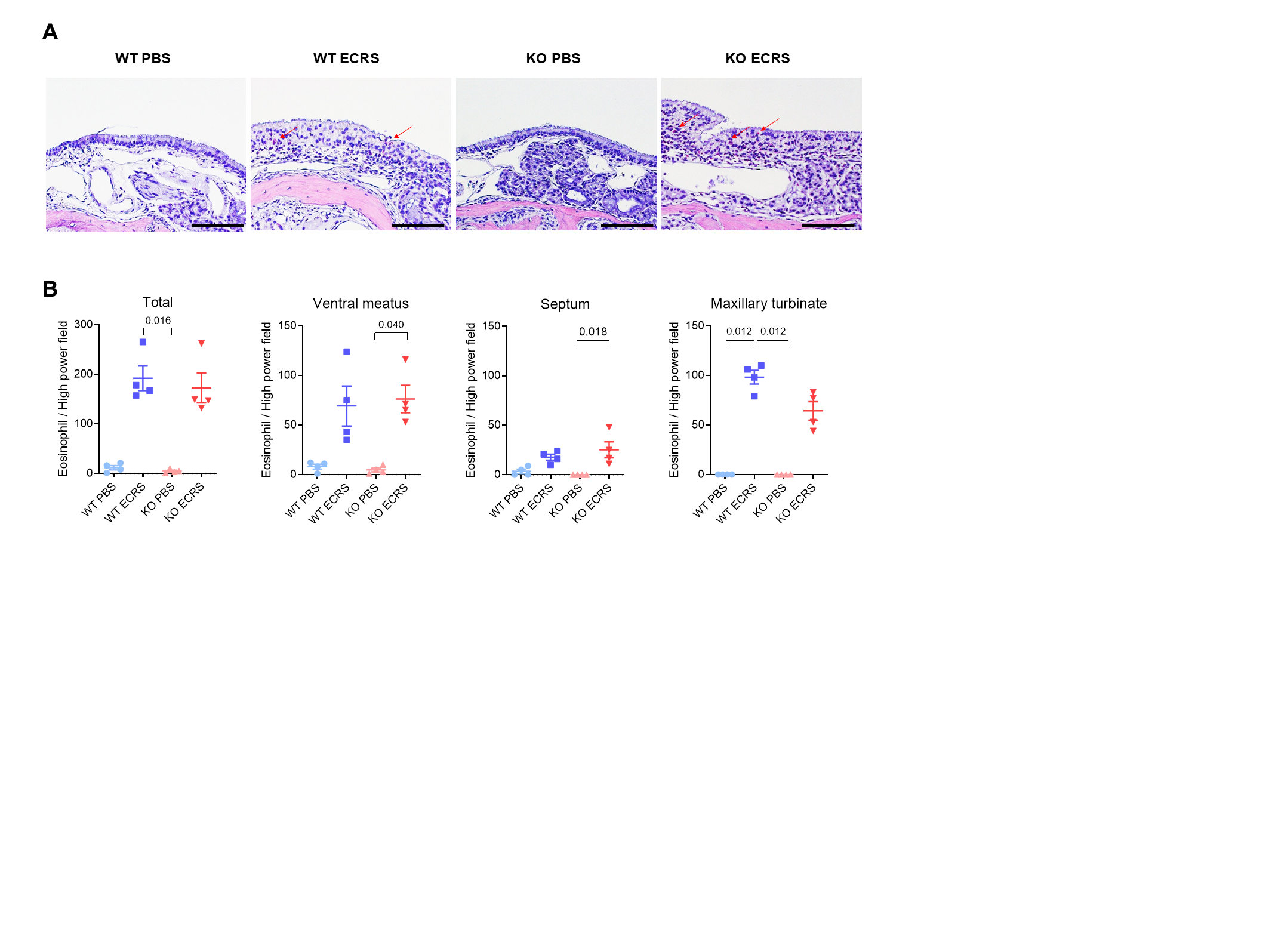

Supplement: Supplementary file 3 [file Image3.tif]

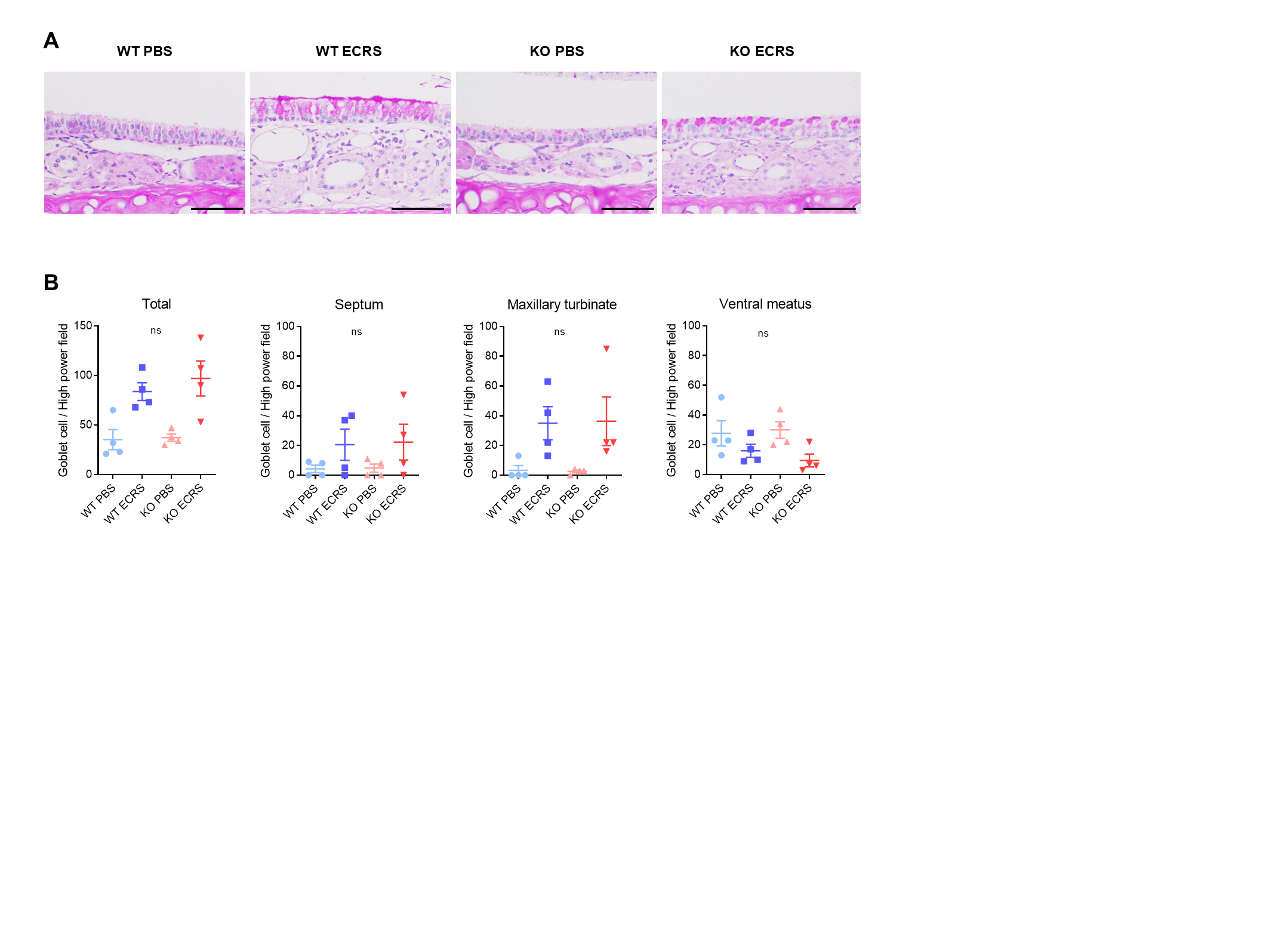

Supplement: Supplementary file 4 [file Image4.tif]

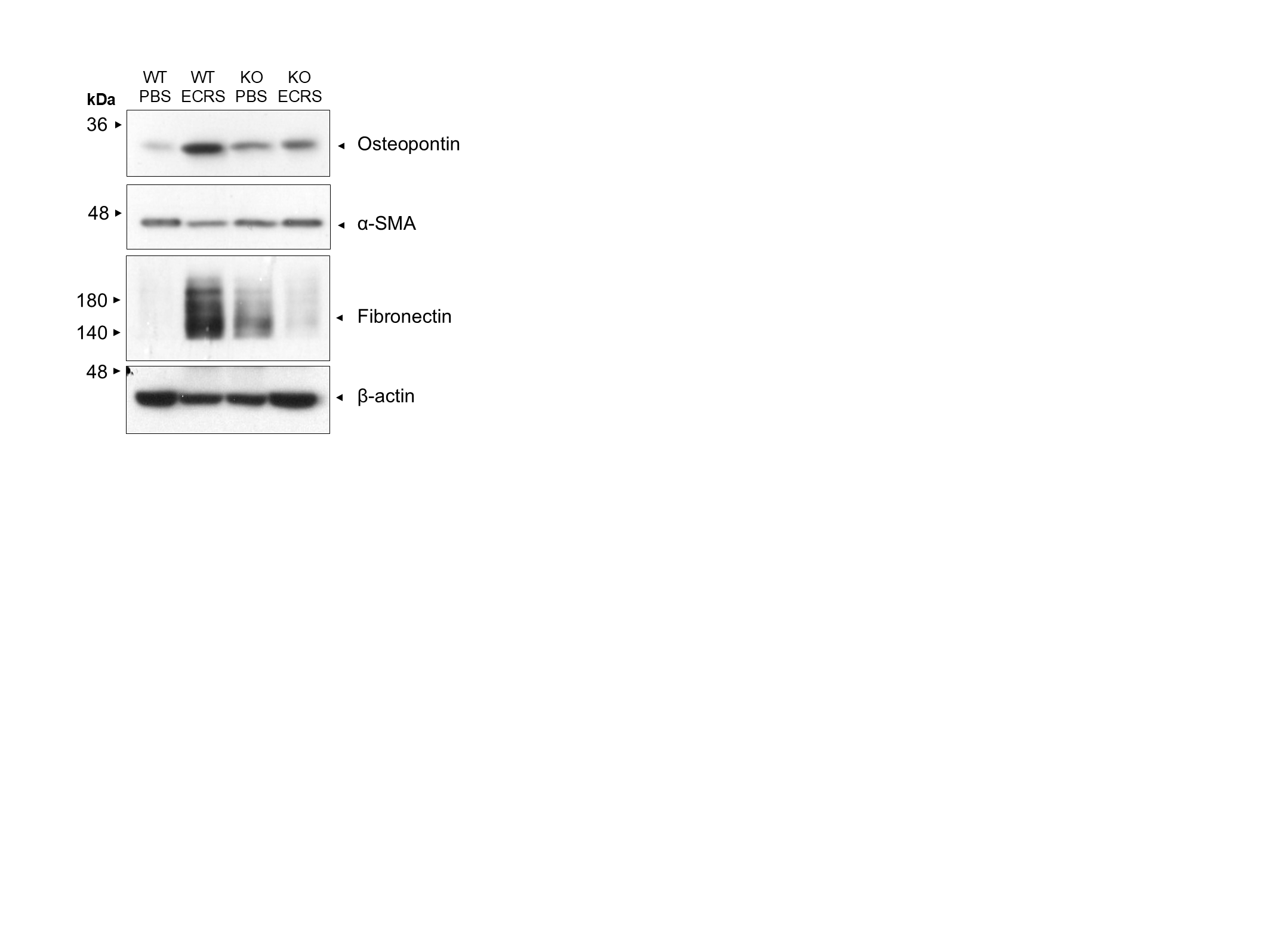

Supplement: Supplementary file 5 [file Image5.tif]

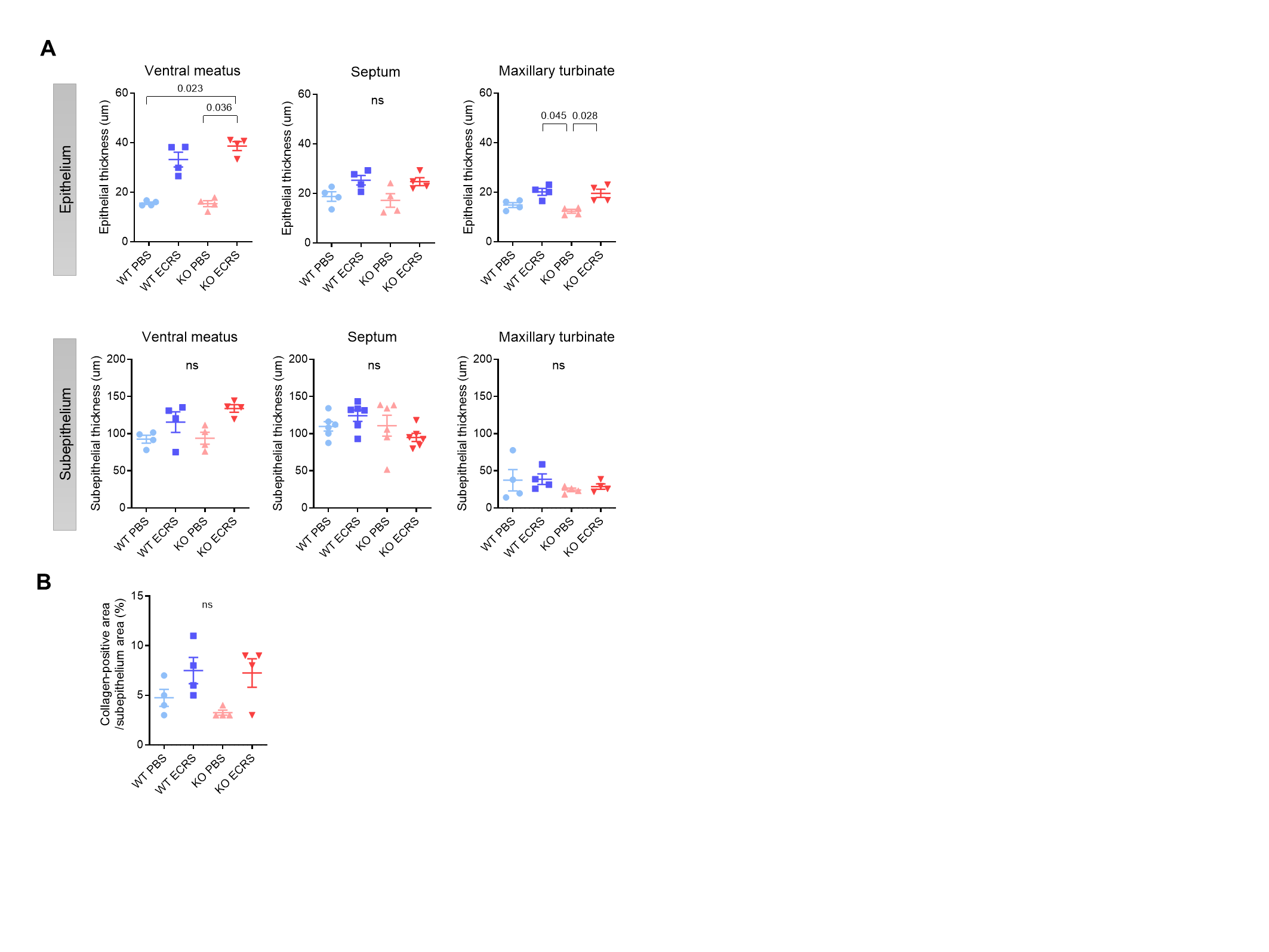

Supplement: Supplementary file 6 [file Image6.tif]

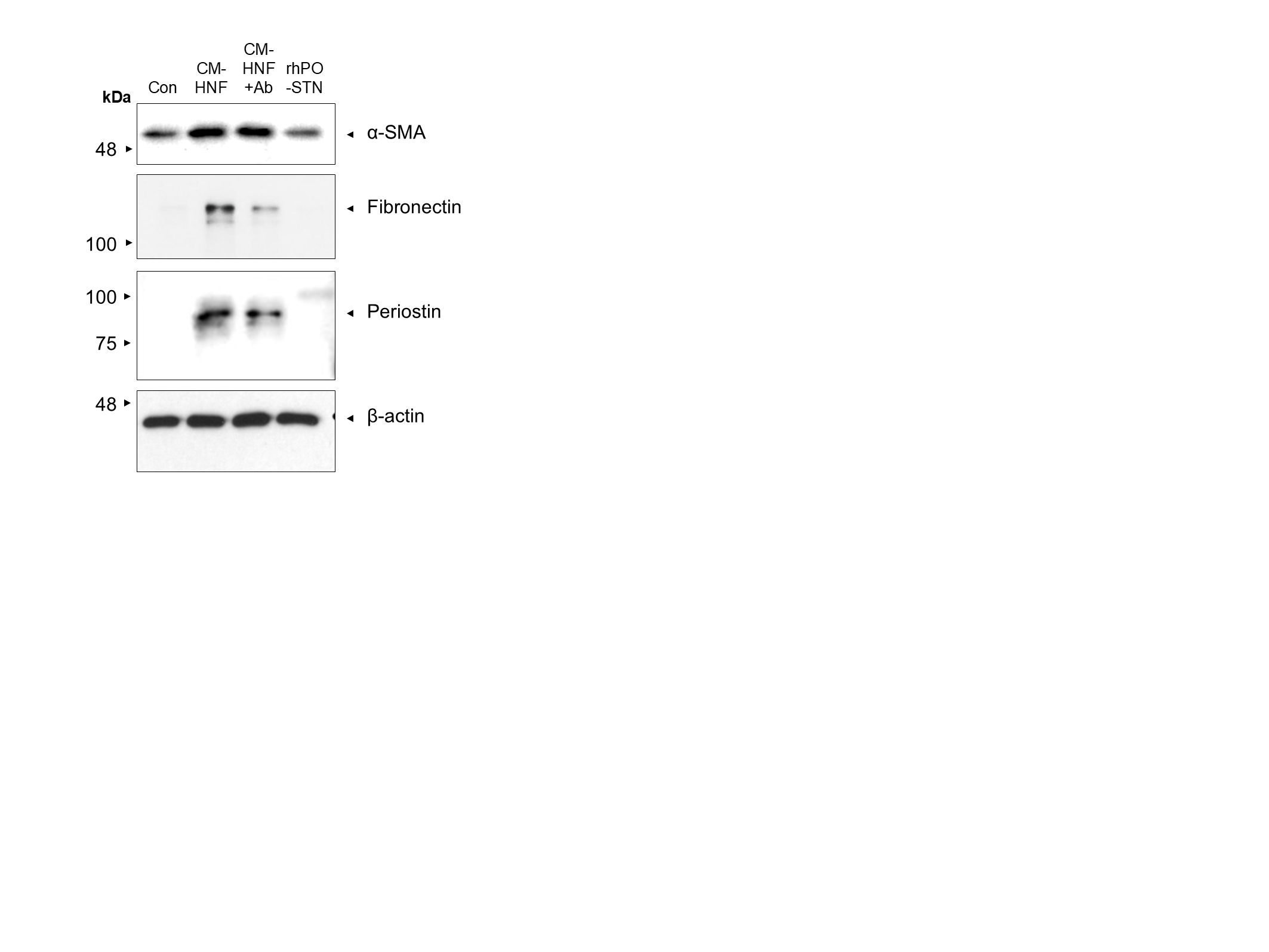

Supplement: Supplementary file 7 [file Image7.tif]
