# Supplementary material for: Inflammation-driven periostin in ECRS has contrasting effects on tissue structural integrity and osteitis
Source: Front Immunol. 2025 Jun 18;16:1596746. doi: 10.3389/fimmu.2025.1596746 (PMC12213678; doi:10.3389/fimmu.2025.1596746)
Supplement: Supplementary file 8 [file Table1.docx]

# Supplementary Tables

**SUPPLEMENTARY TABLE S1**. Demographic and clinical information of patients.

| **Subject** | **Disease status** | **Sex** | **Age** | **Allergic**  **sensitization** | **Lund-Mackay**  **CT score** |
| --- | --- | --- | --- | --- | --- |
| **number** |  |  | **(years)** |  |  |
| S1 | Control | M | 21 | Positive | N/A |
| S2 | Control | F | 65 | Negative | N/A |
| S3 | Control | M | 64 | Negative | N/A |
| S4 | ECRS | M | 45 | Positive | 12 |
| S5 | ECRS | M | 35 | Negative | 9 |
| S6 | ECRS | M | 54 | Positive | 10 |
| S7 | ECRS | M | 64 | Negative | 16 |
| S8 | ECRS | M | 53 | Positive | 20 |
| S9 | ECRS | M | 49 | Negative | 19 |
| S10 | ECRS | M | 59 | Positive | 11 |
| S11 | ECRS | M | 20 | Negative | 17 |
| S12 | ECRS | M | 40 | Negative | 15 |
| S13 | ECRS | F | 70 | Negative | 18 |
| S14 | non-ECRS | F | 43 | Negative | 6 |
| S15 | non-ECRS | M | 59 | Negative | 8 |
| S16 | non-ECRS | M | 63 | Positive | 3 |
| S17 | non-ECRS | F | 37 | Negative | 4 |
| S18 | non-ECRS | M | 48 | Negative | 10 |
| S19 | non-ECRS | F | 26 | Negative | 4 |
| S20 | non-ECRS | F | 38 | Positive | 6 |
| S21 | non-ECRS | M | 16 | Positive | 12 |
| S22 | non-ECRS | F | 42 | Negative | 2 |
| S23 | non-ECRS | M | 35 | Positive | 13 |
| S24 | non-ECRS | F | 60 | Negative | 4 |
| S25 | non-ECRS | F | 65 | Positive | 2 |
| S26 | non-ECRS | F | 63 | Negative | 4 |

ECRS: eosinophilic chronic rhinosinusitis, non-ECRS: non-eosinophilic chronic rhinosinusitis, CT: computed tomography, N/A: not applicable.
