# Supplementary material for: Inflammation-driven periostin in ECRS has contrasting effects on tissue structural integrity and osteitis
Source: Front Immunol. 2025 Jun 18;16:1596746. doi: 10.3389/fimmu.2025.1596746 (PMC12213678; doi:10.3389/fimmu.2025.1596746)
Supplement: Supplementary file 9 [file Table2.docx]

# Supplementary Tables

**SUPPLEMENTARY TABLE S2**. Cell culture media and compositions.

| Name | Media | Supplements |
| --- | --- | --- |
| HNEC subculture medium | BEBM (Lonza) | BEGM™ SingleQuot™ Kit Supplements & Growth Factors (Lonza), EGF (Corning), BSA (GenDEPOT) |
| HNEC ALI culture medium | BEBM (Lonza), DMEM (Lonza) (1:1) | BEGM™ SingleQuot™ Kit Supplements & Growth Factors (Lonza), BSA (GenDEPOT). |
| Fibroblast culture medium | RPMI 1640 (Lonza) | FBS (Lonza), sodium pyruvate (Gibco), L-glutamine (Gibco), NEAA (Gibco), pen-strep (Gibco) |
| Fibroblast serum-free medium | RPMI 1640 (Lonza) | BSA (GenDEPOT), sodium pyruvate (Gibco), L-glutamine (Gibco), NEAA (Gibco), pen-strep (Gibco) |
| MG63 culture medium | EBSS (Lonza) | L-glutamine (Gibco), NEAA (Gibco), FBS (Lonza) |
| MG63 osteogenic medium | EBSS (Lonza) | L-glutamine (Gibco), NEAA (Gibco), ascorbic acid (Sigma), β-glycerophosphate (Sigma). |

BSA: Bovine Serum Albumin, EGF: Epidermal Growth Factor, FBS: Fetal Bovine Serum, NEAA: Non-Essential Amino Acids Solution, pen-strep: penicillin–streptomycin
