# Supplementary material for: Inflammation-driven periostin in ECRS has contrasting effects on tissue structural integrity and osteitis
Source: Front Immunol. 2025 Jun 18;16:1596746. doi: 10.3389/fimmu.2025.1596746 (PMC12213678; doi:10.3389/fimmu.2025.1596746)
Supplement: Supplementary file 10 [file Table3.docx]

# Supplementary Tables

**SUPPLEMENTARY TABLE S3**. Experimental reagents and materials.

| Reagent | Supplier | Catalog No. | Application |
| --- | --- | --- | --- |
| D.pteroyssinus | GREER LABORATORIES | NC9756554 | ECRS mouse model |
| *Aspergillus fumigatus* | GREER LABORATORIES | NC1677927 | ECRS mouse model |
| *Alternaria alternata* | GREER LABORATORIES | NC1620293 | ECRS mouse model |
| Protease (*S. Aureus*) | Abnova | P5254 | ECRS mouse model |
| Anti-fibronectin (FN1) antibody | Abcam | ab2413 | Immunofluorescence, Wetern blot |
| Periostin/OSF-2 Isoform 2 Antibody | R&D Systems | AF2955 | Immunofluorescence |
| Periostin/OSF-2 Isoform 2 Antibody | R&D Systems | AF3548 | Nuetralization |
| Anti-Osteopontin antibody | Abcam | ab218237 | Immunofluorescence, Wetern blot |
| Anti-alpha smooth muscle Actin antibody | Abcam | ab5694 | Immunofluorescence, Wetern blot |
| Anti-Periostin antibody | Abcam | ab14041 | Wetern blot |
| Anti-Periostin antibody | Abcam | ab152099 | Wetern blot |
| β-Actin (C4) Antibody | Santacruz | sc-47778 | Wetern blot |
| Alkaline Phosphatase Staining Kit | Abcam | ab284936 | ALP staining |
