# Supplementary material for: Inflammation-driven periostin in ECRS has contrasting effects on tissue structural integrity and osteitis
Source: Front Immunol. 2025 Jun 18;16:1596746. doi: 10.3389/fimmu.2025.1596746 (PMC12213678; doi:10.3389/fimmu.2025.1596746)
Supplement: Supplementary file 11 [file Table4.docx]

# Supplementary Tables

**SUPPLEMENTARY** **TABLE S4**. Primers for quantitative real-time PCR.

| Gene (species) | Sequence |
| --- | --- |
| *Postn* (mouse)-F | 5′-CAG CAA ACC AC T TTC ACC GAC C-3 |
| *Postn* (mouse)-R | 5′-AGA AGG CGT TGG TCC ATG CTC A-3′ |
| *POSTN* (human)-F | 5′-GCT ATT CTG ACG CCT CAA AAC T-3′ |
| *POSTN* (human)-R | 5′-AGC CTC ATT ACT CGG TGC AAA-3′ |
| *ALP* (human)-F | 5′-TTG GGC AGG CAA GAC ACA-3′ |
| *ALP* (human)-R | 5′-GAA GGG AAG GGA TGG AGG AG-3′ |
| *OCN* (human)-F | 5′-ACC ATC TTT CTG CTC ACT CTG CT-3′ |
| *OCN* (human)-R | 5′-CCT TAT TGC CCT CCT GCT TG-3′ |

POSTN: periostin, ALP: Alkaline Phosphatase, OCN: Osteocalcin.
